# Archaeobotanical evidence reveals the nature of cereal agriculture at 8th- and 9th-century ad Sedgeford, East Anglia, UK

**DOI:** 10.1007/s00334-026-01091-w

**Published:** 2026-05-19

**Authors:** Hannah Caroe, Amy Bogaard, Elizabeth Stroud

**Affiliations:** 1 Present Address: School of Archaeology, University of Oxford, 1 South Parks Rd, Oxford, OX1 3TG UK; 2 Santa Fe Institute, NM 87501 Santa Fe, USA

**Keywords:** Early Medieval, England, Malting, Crop husbandry, Crop rotation

## Abstract

Sedgeford’s multi-feature malting complex, dated to the 8th to 9th century ad, is the earliest known of the early medieval era in England (the approximately contemporaneous, sizeable malting kiln at Higham Ferrers not being part of a larger complex). Three complementary approaches are here used to reveal cultivation practices for cereal grains (predominantly rye and free-threshing wheat) malted at Sedgeford. Specifically, these are used to determine evidence for the three elements of the medieval ‘mouldboard plough package’: namely, ‘extensive’ cultivation, the use of a mouldboard plough and crop rotation. In terms of cultivation intensity, functional weed ecology (FWE) reveals an extensive crop husbandry regime (i.e. maintaining low levels of fertility), whilst stable nitrogen isotope analyses indicate low levels of manuring. FWE also reveals high levels of disturbance, consistent with mouldboard plough use. Finally, all three approaches are used to assess evidence for crop rotation. First, correspondence analyses of the assemblage are used to explore seasonality in crop cultivation. Results of correspondence analyses, together with those from FWE and carbon and nitrogen stable isotope analyses all provide evidence for early crop rotation in fields supplying the malting complex. In sum, evidence suggests that all three elements of the medieval ‘mouldboard plough package’ were present at 8th and 9th century ad Sedgeford.

**Supplementary Information:**

The online version contains supplementary material available at 10.1007/s00334-026-01091-w.

## Introduction

Peoples of early medieval England drank beer on an ‘oceanic’ scale (Finberg 1972, p 422). Whilst high levels of beer consumption were the norm across much of early medieval Europe, the English in particular were known for their heavy drinking (Knowles 1963, p 465; Thomas 2003, p 301). However, as noted by Carruthers and Hunter Dowse (2019, p 107), corresponding archaeobotanical evidence for beer production and consumption in the period has been conspicuously lacking. Also lacking has been thorough archaeobotanical characterisation of the agricultural regimes used to produce grain for beer in the era: a vital part of beer-making and of the wider story of agriculture in early medieval England.

This paper’s specific focus is on understanding how crops supplying a securely-identified malting complex dated to the 8th to 9th centuries (c.) ad were cultivated, in the context of questions about theorised transformation in agricultural practice during the 7th to 9th c. ad (so-called *mid Anglo-Saxon* period) in England (cf. McKerracher 2018). The study assemblage derives from the unique, large-scale malting complex at Sedgeford, East Anglia, UK. Malting, the first stage of beer production, is conventionally evidenced archaeobotanically by abundant and widespread germinated cereal grains (e.g. Lodwick 2017). An earlier paper (Caroe 2022a) gave an overview of the assemblage, detailing evidence of grain germination and other factors confirming that Sedgeford’s is indeed a malting complex, as well as emphasising the wider context in terms of beer production and consumption.

The so-called medieval agricultural ‘revolution’ in England (e.g. White 1940; Williamson 2018) has recently been investigated by an extensive multi-proxy interrogation of bioarchaeological remains by the University of Oxford’s FeedSax project (Hamerow et al. 2019, 2025; McKerracher and Hamerow 2022). Rather than identifying a single period of ‘revolutionary’ change, changes in agricultural practice in England between ca. ad 700–1300 were piece-meal and regionally varied, over several centuries, ‘punctuated by periods of innovation and rapid change’ (Hamerow 2022, p 24), including the late 7th to 9th c. ad (Hamerow 2007; Rippon 2010; McKerracher 2018; Williamson 2018).

FeedSax’s research has identified three features commonly associated with changes in early medieval agriculture – extensification, use of the mouldboard plough and crop rotation – dubbing this the ‘mouldboard plough package’ (Hamerow 2022). Extensification is defined as increase in crop productivity achieved through expanding the land area under cultivation, associated with a decline in inputs of manure and labour (weeding and tillage) per areal unit of land (Bogaard et al. 2016; Hamerow 2022). Research using FWE (see below) implies a shift from relatively high (intensive) to relatively low input (extensive) arable farming across England from the 8th until the 14th c. ad (Hamerow et al. 2025, pp 102–103), continuing a trend already apparent in the Romano–British era (e.g. Lodwick 2022).

The mouldboard plough permitted expansion of cultivated land onto heavy clay soils. A distinctive mouldboard plough coulter dated to the 7th c. ad has been recovered at the royal monastic site of Lyminge, Kent (Thomas et al. 2016). This evidences early use at isolated locations, but is (so far) by some margin the earliest known in England, and zooarchaeological evidence for pathologies in cattle foot-bones indicating heavy traction suggest that mouldboard plough use was itself rare until the mid-9th c. ad (Holmes 2022). Further, there is minimal pre-10th c. ad documentary evidence for the mouldboard plough (Holmes 2022). However, the lack of earlier documentary records may be due to the dearth of textual evidence from the era (Holmes 2022).

‘Classic’ crop rotation involved two or three fields, with one remaining fallow while the other(s) were cultivated. In three-field rotation, one field is sown with an autumn-sown crop (generally wheat, sometimes rye or barley), and one with a spring-sown (often barley or oats, occasionally wheat). In subsequent years, use rotated (Hall 2014, p 36). FeedSax research does not evidence the country-wide 7th to 9th c. ad shift to this field system that some have advocated (e.g. White 1940).

We here use three methodologies to examine evidence for crop rotation and other elements of the ‘mouldboard plough package’ in the fields supplying Sedgeford’s 8th and 9th c. ad malting complex. Combining evidence from FWE, carbon and nitrogen stable isotope analyses and correspondence analysis, we find evidence for all three elements of the mouldboard plough package in use at Sedgeford in this era.

## The study site

**Fig. 1:**
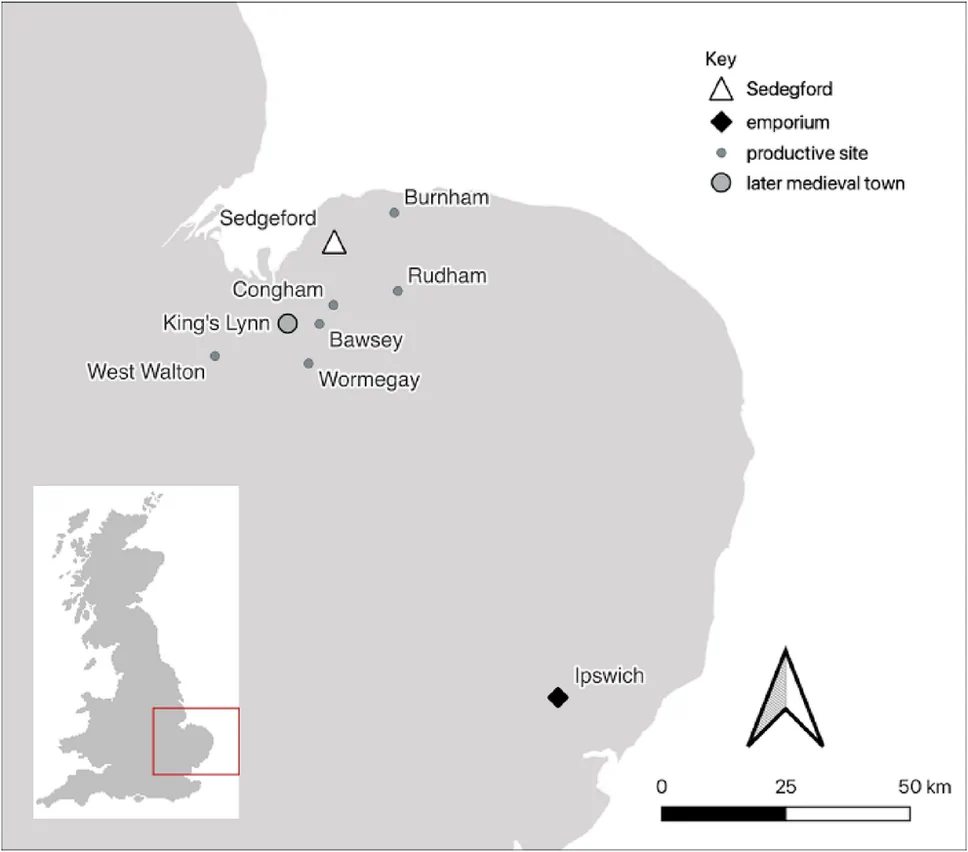
Sedgeford and significant local sites. The map includes ‘productive sites’ of NW Norfolk, as identified by Rogerson (2003). These are locations which have produced abundant metal-detector finds, sometimes thought to represent rural market sites (e.g. Ulmschneider 2000). Contains Ordnance Survey Open Data © Crown copyright and database right 2017, under the Open Government licence. Map created with QGIS (www.qgis.org)

**Fig. 2:**
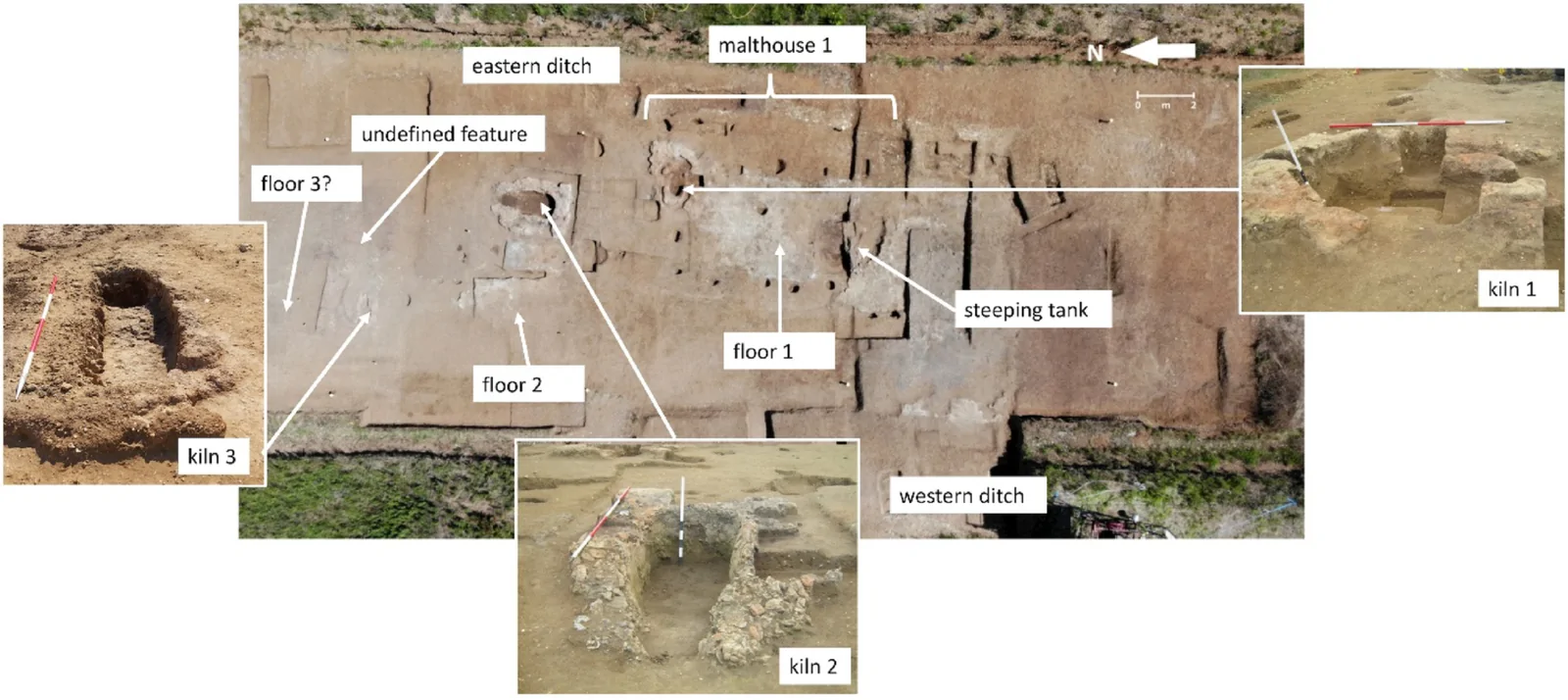
Aerial photograph of the malting complex, with primary features highlighted, and inset photographs of kilns (Image: Ian Drummond/SHARP 2019. Shared with kind permission)

The archaeological site of Sedgeford lies about six km inland from the coast of northwest Norfolk, in East Anglia, UK (Fig. 1). The regional bedrock is Upper Cretaceous Middle Chalk, and the soil type is ‘shallow, lime-rich’ (Chatwin 1961, p 32). Excavation at Sedgeford since 1996 has revealed an extensive early medieval cemetery and a likely settlement area, the latter apparently occupied from the 7th to 11th c. ad; two recent radiocarbon dates here derive from late 8th to late 9th c. ad deposits (Faulkner and Blakelock 2020; McKerracher 2023a). Excavation of an area to the south of the original site (trench 23) revealed a set of features including ovens/kilns and floors, with abundant charred plant material, surmised to represent together a cereal-processing complex. Archaeobotanical assay revealed that many charred grains in this part of the site had germinated prior to charring, fostering the hypothesis that the features denote a malting complex (Wolff 2017). At least three kilns have been clearly identified at the site (several potential kilns remain to be fully excavated), along with a purported steeping tank and a set of clay floors (Fig. 2), features arguably associated with the three stages of malting (steeping, germination and kilning) and thus consistent with a ‘malting complex’ designation.

Rye grains from each of kilns 1, 2 and 3 were radiocarbon dated in 2019. Dates obtained using Bayesian modelling are, respectively, cal ad 748–770, 734–775 and 772–819 (McKerracher 2023b). Significantly, these reveal that Sedgeford’s is the earliest known malting complex in early medieval Britain and Ireland. Approximately contemporaneous finds including the sizeable malting kiln at Higham Ferrers (Northamptonshire), and potential malting pits at South Hook (Pembrokeshire), are, in each case, not part of a multi-feature complex including steeping tank(s) and germination floor(s) (Hardy et al. 2007; Crane and Murphy 2010). A review of malting buildings in England reveals that excavated malting kilns dating from prehistory until the later medieval era are very rarely accompanied by recognised malting floors or steeping cisterns (Patrick 2023, p 5).

## Materials and methods

Sediment samples for extraction of archaeobotanical material from trench 23 were initially selected according to ‘judgment’ sampling (M. Jones 1991) i.e. where dark organic remains were visible, or charred plant remains were expected. From 2019, additionally, an ‘interval’ (M. Jones 1991) sampling grid-system was implemented in an area surrounding kiln 3. Sample volume varied from 5 to 70 L.

Plant material was recovered using an Ankara-style flotation device (French 1971). Estimated richness, plant taxa diversity and context information for the samples were used to select samples to analyse. Altogether, including 15 samples from the gridded area, 55 samples from the malting complex have been analysed.

Samples were sorted using a stereomicroscope in the School of Archaeology, University of Oxford, with plant material identified using the lab’s reference collection and relevant literature (Jacomet 2006; Stace 2010; Cappers et al. 2013). Latin nomenclature follows Stace (2010). All plant material in the assemblage is charred.

The minimum number of individual plant items (MNI) was determined by counting specific diagnostic zones (G. Jones 1991). For cereal grains the frequency of both apical and embryo ends per sample was counted, and the higher figure recorded. Most weed seed taxa occurred infrequently in each sample and were scored even when fragmented. Where several fragments co-occurred, the MNI was estimated.

### Functional weed ecology

Two sets of FWE analyses were conducted. The first pertains to intensity of agricultural regime, where intensity relates to both fertility (which may be associated with the level of manuring) and, to a lesser extent, disturbance caused by weeding/tillage. The second analysis relates to disturbance (level of weeding/tillage) alone. First, the weed spectrum from Sedgeford was classified by a discriminant analysis function using an existing ‘extensive/intensive’ FWE model. The model contrasts two sets of weed flora from modern surveys in known extensive and intensive agricultural regimes from Haute Provence, France and Asturias in Spain, respectively (Charles et al. 2002; Bogaard et al. 2016). Secondly, the Sedgeford weed set was classified by a different discriminant analysis function within a model contrasting floras from high- and low-disturbance modern regimes, based on modern weed surveys at Laxton, (Nottinghamshire, UK) and Highgrove Home Farm (Gloucestershire, UK) (Hamerow et al. 2020; Bogaard et al. 2022). The disturbance model contrasts low disturbance unploughed ‘sykes’ at Laxton with high disturbance regimes: fallow fields in use as part of a rotation scheme at Laxton and arable fields at both Laxton and Highgrove (Hamerow et al. 2020; Bogaard et al. 2022). ‘Syke’ is the name given to an occasionally grazed, cut once-yearly, untilled patch of hay meadow between or on the edges of other (‘open’) fields at Laxton, which is not treated with herbicide (Hamerow et al. 2020 p.13).

**Fig. 3:**
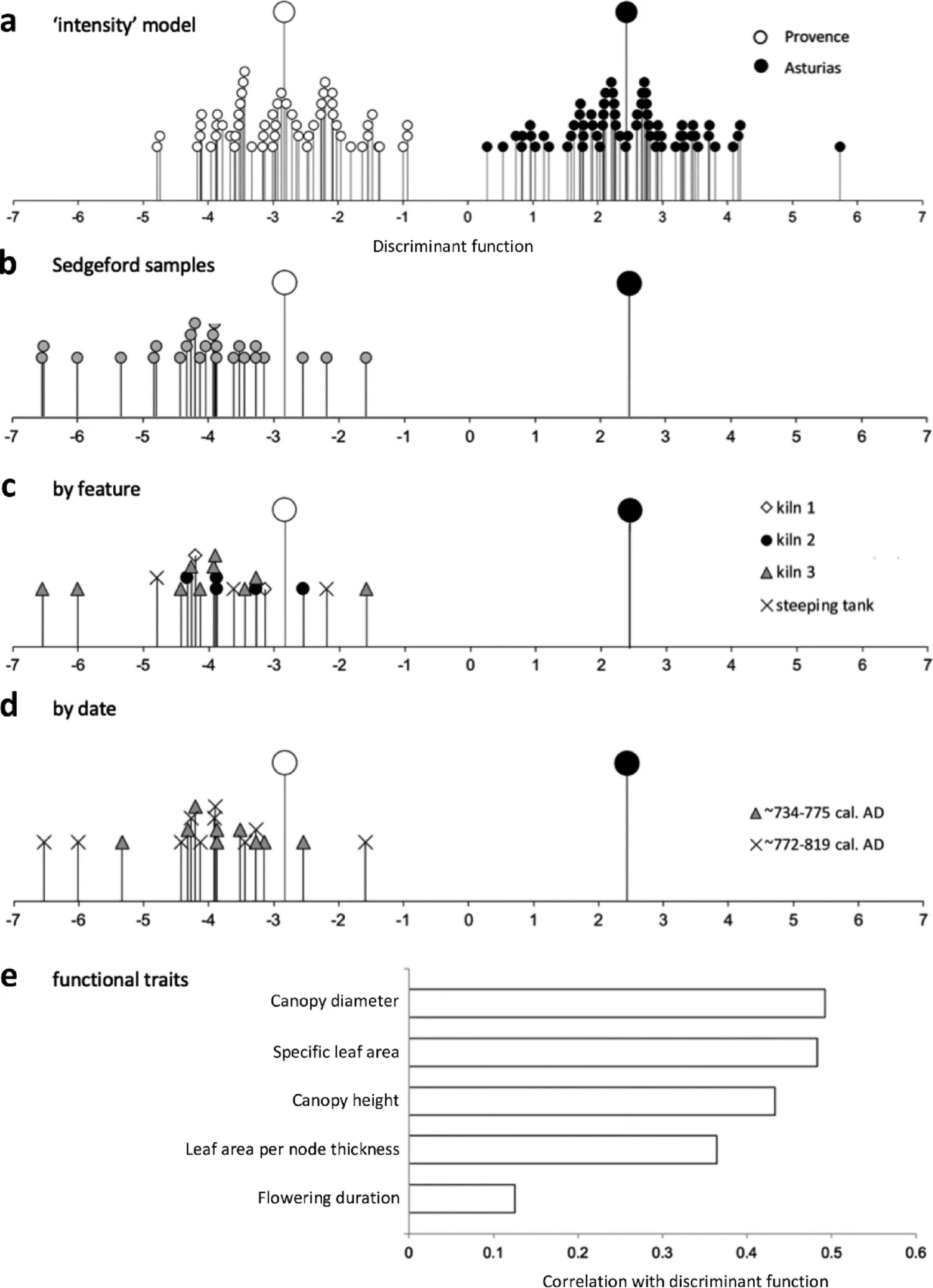
‘Intensity’ discriminant analysis plots. **a** Scores of modern arable plots along the discriminant function extracted to separate low- (white) and high-intensity (black) crop cultivation regimes. Larger symbols indicate group centroids (average scores). **b** Relationship between units from the Sedgeford assemblage and the discriminant function used to distinguish high- and low-intensity modern regimes (centroids as before). **c** Replica of plot b) with episodes coded by malting complex feature. **d** Replica of plot b) with episodes coded by date range. **e** Correlations between functional trait scores used as discriminating variables and the discriminant function. Both plots (a) and (e) are reproduced with permission from Bogaard et al. (2016, p 66, Figs. 6b and 7b)

**Fig. 4:**
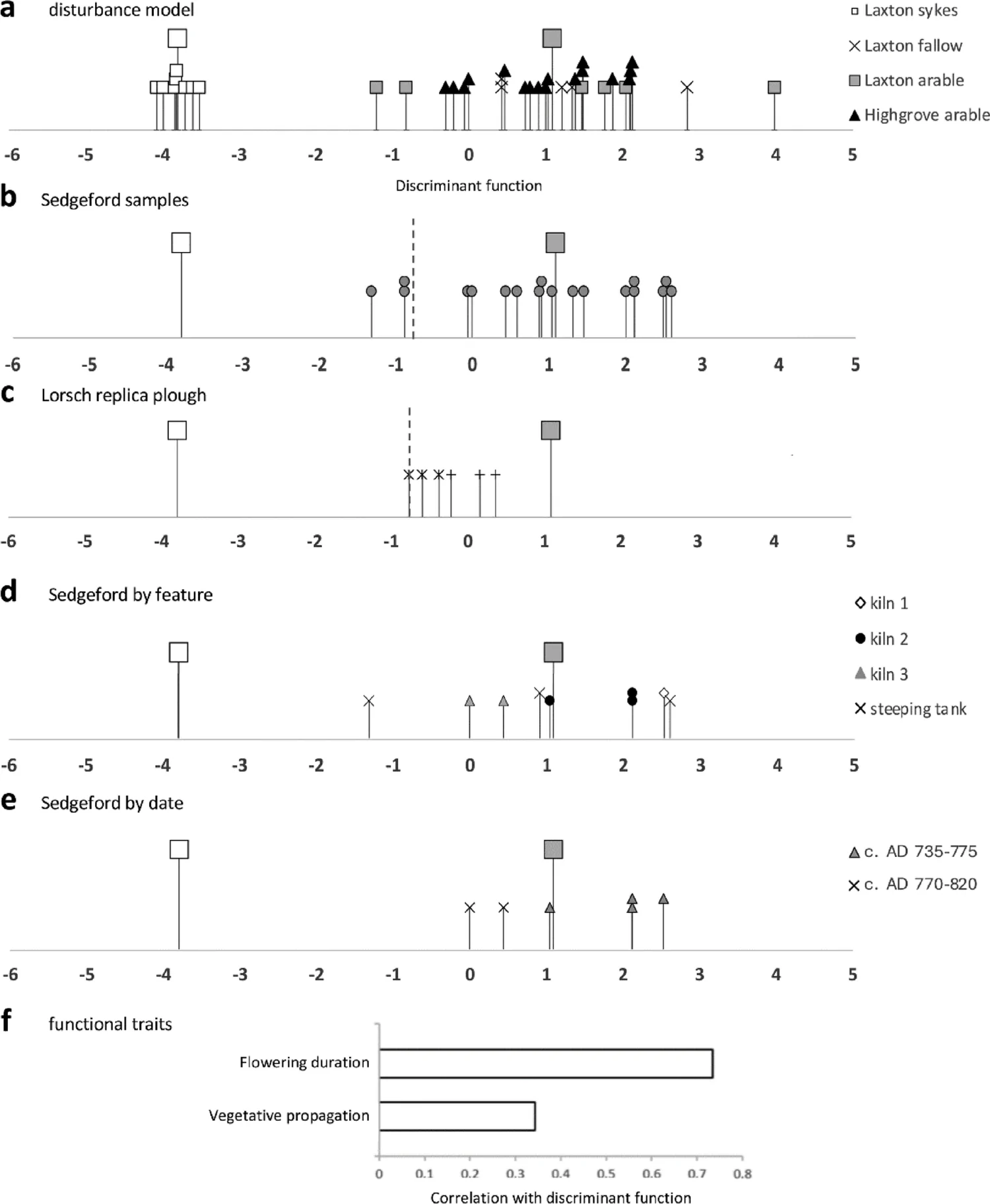
Disturbance discriminant analysis plots. **a** Relationship of low-disturbance regime (white squares) and of high-disturbance regimes (other symbols) with the discriminant function. Larger symbols are group centroids (average scores). **b** Relationship between units from the Sedgeford assemblage and the discriminant function extracted to distinguish low-disturbance from high-disturbance regimes. Larger symbols are group centroids. **c** Relationship of experimental mouldboard-ploughed fields from Lorsch with the extracted discriminant function, centroids as before (shared with permission from Bogaard et al. (2022) Figs. 13, 14, 15). **d** Same plot as b) with units coded by malting complex feature. **e** Same plot as b) with units coded by date range. In plots b) and c), the ‘Lorsch baseline’ is shown as a dashed line. **f** Correlations between functional trait scores used as discriminating variables and the discriminant function. Both plots (a) and (f) are reproduced with kind permission from Hamerow et al. (2020, p 599, Fig. 8a and f).

The analysis is based on recording functional traits in modern weed plants which correlate with species’ potential under variable growing conditions (Charles et al. 1997; Bogaard et al. 2001; Jones 2002). The two FWE models applied here use different sets of functional traits to achieve the best separation of the modern regimes. Traits which best distinguish more- and less-fertile regimes (included in the intensity model) are plant canopy height, canopy diameter, specific leaf area and the ratio of leaf area per node to fresh leaf thickness (Bogaard et al. 2016) (Fig. 3e). The length of the flowering period and – for perennial plants – capacity for vegetative regeneration best distinguish between high- and low-disturbance regimes (Bogaard et al. 2022) (Fig. 4f). In each case, in the ‘discrimination phase’, a discriminant analysis model was created, using a linear equation which optimally separated the two sets of known regime attribute data. In the subsequent ‘classification phase’, functional trait data based on modern plant analogues of weed species represented as seeds in the Sedgeford samples were used to classify each sample into one of the two contrasting known groups, according to the linear discriminant equation previously extracted to maximise separation between the contrasting modern regimes.

With reference to the disturbance model, a reconstructed ox-drawn mouldboard plough has recently been used as part of a three-field rotation system at the Lauresham Open-Air Laboratory for Experimental Archaeology in Lorsch, Germany (Bogaard et al. 2022; Kropp 2022; Stroud et al. 2024). FWE was conducted on the weed flora from this field study. Bogaard et al. (2022) devised a ‘Lorsch baseline’ representing the lowest expected discriminant score for fields tilled with a mouldboard plough (Fig. 4c), and this is compared to Sedgeford data here.

Only Sedgeford samples with at least 10 weed seeds identified to species level were included in the analyses (on this basis, sample 17018 was excluded). Further, spatially proximate samples with similar composition were grouped, to give 26 distinct ‘behavioural episode’ units. Grouping samples into ‘behavioural episodes’ was undertaken for the purposes of FWE alone. Stable isotope and correspondence analyses were conducted using ungrouped samples. This analysis is equivalent to the workflow now formalised in the ‘R’ package, WeedEco (Models 1 and 3) (Stroud et al. 2024) but was conducted before this package was available. Analysis was conducted using IBM SPSS version 27.

### Stable isotope analysis

FWE results are complemented by additional independent lines of evidence for revealing crop husbandry methods, e.g. stable isotopic analysis (Bogaard et al. 2016; Stroud et al. 2021). Both stable carbon and nitrogen isotope analyses were conducted. The ratio of stable carbon isotopes, ^13^C to ^12^C (δ^13^C), in preserved grains can indicate levels of soil water availability for crops (e.g. Wallace et al. 2013; Styring et al. 2017; Stroud 2022), whilst archaeological grains’ ratio of stable nitrogen isotopes, ^15^N to ^14^N (δ^15^N), can be used to investigate soil nitrogen isotopic ratio, and thus the possibility of manuring (e.g. Bogaard et al. 2007; Styring et al. 2017; Stroud 2022).

Reduced enzymatic discrimination against the heavier ^13^C isotope during plant photosynthesis when stomatal pores are closed at times of water stress, means a plant’s carbon stable isotope ratio correlates with its water status (e.g. Farquhar et al. 1989; O’Leary 1993). δ^13^C values can aid elucidation of cultivation methods even in temperate climates such as the UK’s, where plant water stress is rare, e.g. by revealing whether sets of crops were grown in similar water availability conditions, potentially consistent with crop rotation (Hamerow et al. 2025, p 129). Where crop δ^13^C values differ significantly and consistently between species, indicating different water availability conditions, crop rotation is unlikely to have been in use. There are various possible explanations for crops having been cultivated in similar water availability conditions – one amongst these is crop rotation.

Barley grains have a δ^13^C value of ~ 1–2‰ lower (six-row barley grains ~ 2‰ lower) than free-threshing wheat grains from crops grown with equal water availability (Anyia et al. 2007; Wallace et al. 2013). δ^13^C values are also influenced, inter alia, by soil type, canopy cover, light, temperature, and topography (Heaton 1999; Bogaard et al. 2016).

Turning to stable nitrogen isotopic analysis, factors influencing soil and thus plant tissue δ^13^N values include aridity, salinity, waterlogging and crop manuring (Heaton 1987; Bogaard et al. 2007; Larsson et al. 2019). Preferential volatisation of the lighter ^14^N isotope in manure means that plants grown in manured soil themselves become enriched in ^15^N (Bol et al. 2005; Bogaard et al. 2007). Detecting manuring of fields requires knowledge of the δ^13^N values of plants grown on local unenriched soils (e.g. Stroud et al. 2021); this may be estimated based on δ^13^N values of archaeological wild herbivore bone collagen (Hedges and Reynard 2007; Bogaard et al. 2013) assuming herbivores were foraging on wild rather than crop plants. Nitrogen stable isotope values are available for collagen from two wild herbivores from Anglo-Saxon era East Anglia (two deer, both in Suffolk) (ESM 1 Table S1) (Leggett 2021, p 93). The mean δ^15^N value for the herbivore collagen is 5.9‰. To compare these with cereal grain δ^15^N values, 4‰ was subtracted to compensate approximately for the trophic shift between vegetation and herbivore, and 2.4‰ added to account for the offset between grains (equivalent to seeds or fruits) and rachis (equivalent to leaves or stems), the plant parts generally consumed by herbivores (Fraser et al. 2011; Bogaard et al. 2013). The mean calculated ‘wild herbivore baseline’ thus derived for unmanured grains is 4.3‰. These wild herbivore-baseline derived δ^15^N values should be applied with some caution: the samples originate > 34 miles from Sedgeford; further, red deer can browse in woodlands, (i.e. are not always grazers), potentially reducing their bone collagen δ15N values (Sykut et al. 2021).

Other instances where a wild herbivore baseline has been applied to assess δ^15^N values include at the medieval site of Lyminge in Kent (Hamerow et al. 2025, p 88, Fig. 3.31). A particularly robust use of such baselines is Styring et al. 2017 (see especially p 366 Fig. 3).

Single-grain samples of rye, free-threshing wheat and hulled barley were selected from kilns 1, 2, 3 and the steeping cistern. Additional criteria informing grain selection strategy were as follows. First, visibly germinated grains were excluded, to limit potential confounding effects. Secondly, charring can affect δ^13^C and δ^15^N values (Styring et al. 2013; Nitsch et al. 2015; Stroud et al. 2023a). Grain interiors give the best indication of charring temperature (Stroud et al. 2023a; Vaiglova et al. 2023); grains were halved and those judged to fall outside an optimum charring window (215–300 °C) (Stroud et al. 2023a) excluded. In total 112 single-grain samples were analysed; numbers of grains belonging to each species selected, and their contexts, are listed in ESM 1 Table S2.

Fifteen representative single-grain samples were screened using Attenuated Total Reflection Fourier Transform Infrared Spectroscopy (ATR-FTIR) to identify possible contamination post-deposition. No evidence of contamination with carbonates, nitrates or humic acids was found (ESM 1 Fig.S1), thus pre-treatment was deemed unnecessary.

The samples were analysed using a Sercon 20–22 EA-GSL isotope mass spectrometer at the Oxford University Research Laboratory for Archaeology and the History of Art. In-house standards of cow collagen (COW), seal collagen (SEAL) and Alanine were used to calibrate the data according to the internationally determined scales: VPDB (Vienna Pee Dee Belemnite) for carbon and AIR (Atmospheric nitrogen) for nitrogen (Hoefs 2018, p 34). All standards used have well-characterised isotopic compositions; their means and standard deviations (SDs) are listed in ESM 1 Table S3. 

To understand accuracy, precision and uncertainty, an internal leucine standard and EMA P2 were used as check standards, and every 10th sample was duplicated. Following Szpak et al. (2017), the accuracy (u(bias) of the carbon runs was calculated as ± 0.28‰, and the precision (u(Rw) as ± 0.08‰. Combined total analytical uncertainty for δ13C values was ± 0.29‰. For nitrogen, accuracy was ± 0.40‰ and precision ± 0.20‰. Overall uncertainty for δ^15^N values was calculated as ± 0.45‰ (Szpak et al. 2017). Data were calibrated using the statistical programme R (version 4.1.2), and accuracy, precision and uncertainty calculated using Excel, version 16.58.

Following Stroud et al. (2023b), ‘charring’ offsets were applied to the calibrated values: +0.33‰ for δ^15^N values, and + 0.12‰ for δ^13^C values. Following FeedSax practice (e.g. Stroud 2022), calibrated δ^13^C values were not converted to Δ^13^C, since δ^13^C values for archaeological grains are not here compared with those for modern grains (see e.g. Farquhar et al. 1989).

### Correspondence analysis and seasonality

Correspondence analysis is a means of exploring variability in the composition of samples from an assemblage, by representing these graphically. Correspondence analysis was used to investigate trends in the sowing season of crops supplying the malting complex. Species occurring frequently in the assemblage are represented in the correspondence analysis scatterplots featured here. Those whose datapoints fall close to the graph’s origin are common or ubiquitous in samples from the assemblage. Where species are clustered (spatially proximate) in the plot, this indicates that these regularly co-occur in the samples, consistent with a tendency to grow together. Where species are spatially dispersed in the plot, the reverse is true (Bogaard 2004, pp 92–94).

Correspondence analysis plots were created using CANOCO version 5.0 (ter Braak and Smilauer 2012). CANOCO generates for each analysis four axes, with axis 1 accounting for the greatest variation. In all plots shown here the horizontal axis is axis 1, and the vertical, axis 2. It is possible, using CANOCO, to apply mathematical ‘transformations’ to the correspondence analysis values. A square-root transformation, applied in one plot here, can help data to fit the assumptions of correspondence analysis better by reducing skew and ‘stabilising’ variance in the data (Bartlett 1936; Greenacre 2009). Table 1 summarises the short names allotted to each taxon included in the correspondence analyses, as displayed in the scatterplots.

**Table 1:** Annual weed species types based on flowering onset and duration, with associated crop sowing regime, (after Bogaard et al. 2001 Table 3 and McKerracher 2019 Table 27)

Type	Flowering onset	Flowering duration (months)	Competitive advantage in…
Early/short	Jan.–Jun.	1–3	Autumn-sown fields
Late	Jul.–Dec.	1–5	Spring-sown fields
Long	Jan.–Jun.	> 5	Spring-sown fields
Intermediate	Apr.–Jun.	4–5	Autumn and spring-sown fields

Data in the correspondence analyses conducted here are additionally coded by seasonality. We here use correspondence analysis to infer the season of sowing for crops based on associations with weeds whose ecological traits make them more likely to co-occur with spring or autumn-sown cereals (cf. McKerracher 2019, pp 96–127). Weed ecological traits which best reveal crop seasonality are timing of flowering onset and flowering duration (Bogaard et al. 2001). Annual weed species whose germination and flowering time is late in the year or of long duration (setting seed after ploughing for spring sowing) are advantaged among spring sown crops. Species which germinate and flower early and for a short time flourish (being undisturbed) among autumn-sown fields (Bogaard et al. 2001; McKerracher 2019, p 97).

Our assumption that weed species featured in the correspondence analyses grew amongst the crops as genuine crop weeds is based on these surmises: firstly, featured samples were grain-rich and relatively-speaking weed-species poor. Secondly, these were recovered from in situ contexts (rich concentrations of charred grains surrounding malting kilns) not suggestive of secondary mixing. Finally, only weed species occurring above a threshold frequency were incorporated into the analyses.

Samples with < 10 weed seeds were excluded, as were weed species occurring in fewer than 10% of samples. Weed taxa identified only to the family or genus level were also excluded. Eligible Sedgeford weed seeds included two perennials: *Plantago lanceolata* and *Phleum pratense*. Each of these regularly regenerates by seed (as well as through vegetative propagation) hence these were treated as annuals (cf. Bogaard et al. 2001). A total of 53 samples, and 12 weed species (in addition to the cereal taxa) were included in the analyses. The seasonality category assigned to each species is shown in Table 2.

**Table 2:** Weed species from Sedgeford’s malting complex assemblage included in ‘seasonality’ correspondence analysis, with flowering onset/duration class, associated seasonality and short name used in scatterplots

Species	Class	Resultant seasonality	Short name
*Agrostemma githago* L.	Early/short	Autumn	A_gith
*Anthemis cotula* L.	Late	Spring	A_cot
*Atriplex hastata* L. */ patula* L. */ prostrata* Boucher ex. D.C.	Intermediate	N/a	Atri_p
*Bromus arvensis* L. / *hordeaceus* L. / *secalinus* L.	Early/short	Autumn	Bro
*Chenopodium album* L.	Late	Spring	C_alb
*Fallopia convolvulus* (L.) Á.Löve	Late	Spring	F_conv
*Phleum pratense* L.	Early/short	Autumn	P_prat
*Plantago lanceolata* L.	Long	Spring	P_lanc
*Raphanus raphanistrum* L.	Intermediate	N/a	R_raph
*Urtica urens* L.	Intermediate	N/a	U_uren
*Vicia hirsuta* L. (Gray) / *tetrasperma* L. (Schreb.)	Intermediate	N/a	V_h_l

## Results

Examination of the 55 malting complex samples revealed a cereal-rich assemblage, dominated (unusually for Anglo-Saxon England) by rye grains (*Secale cereale* L.) (64%, *n* = 21,814), and secondarily bread wheat (*Triticum aestivum* L.) (28%, *n* = 9,518). Seven per cent (*n* = 2,363) of grains from the assemblage are hulled barley, likely 6-row (*Hordeum vulgare* ssp. *vulgare*) (Caroe 2022a). All three crops occur near ubiquitously in each sample. In total, 61,062 plant items were identified, belonging to 99 taxa/types. Abundances and ubiquity of key taxa are summarised in Table 3.

**Table 3:** Summarising ubiquity and abundance of crop and weed remains in all 55 samples from the malting complex

Plant item	Samples where present		Max. items per sample	Sum of items
	no.	%		
*Cereal grains*				
Rye	55	100	1,391	21,814
Free-threshing wheat	55	100	997	9,518
Hulled barley	53	96.4	267	2,363
Oat	25	46	40	321
*Chaff*				
Rye rachis	22	40	125	699
Bread wheat rachis	12	22	32	124
Hulled barley rachis	15	27	48	204
Weedy/wild oat floret base	2	3.6	8	12
*Weedy/wild seeds*				
Weedy/wild taxa total	55	100	544	9,446
Brome grass	54	98	237	2,766
Corncockle	42	76.4	120	1,183
Black bindweed	36	65.5	480	1,791

The overall proportion of malting complex grains clearly showing signs of germination under standard light microscopy is 17% (*n* = 1,602 of 9,662 assessed); this increases to 46% (*n* = 4,450 of 9,662) following proportional reassignment of grains whose germination status cannot be determined (Caroe 2022a). Only one sample from the malting complex lacked germinated grains. A comparison by way of a ‘control’ with four similarly examined samples from the settlement part of the site, where malting is not believed to have been practiced, revealed that no grains from the settlement show clear evidence for germination (44% had indeterminate germination status).

### Functional weed ecology

#### Intensity of cultivation

Figure 3a displays the weed ecology intensity model (Bogaard et al. 2016). Each smaller symbol represents a single modern arable plot (from either Haute Provence or Asturias), coded according to the type of agricultural regime it represents and plotted along the discriminant function extracted to distinguish between the two groups of high- and low-intensity farming systems (centroids – average scores for each group – are shown). Discriminant function values are calculated using the functional weed trait values per arable plot, entered as discriminating variables in the model, as shown in Fig. 3e. Figure 3b plots the scores of the 26 Sedgeford units, along the same discriminant function. All units cluster at the negative end of the axis and each is classed by discriminant analysis as low intensity. These observations clearly attest that the agricultural regime(s) from which Sedgeford’s assemblage originates was one of low fertility, implying low labour inputs per unit area.

Figure 3c shows no apparent trend when the datapoints are coded by feature within the malting complex. Similarly, there does not seem to be any relationship between the date of the episodes and the discriminant function when datapoints are coded by approximate date range; in other words, there is no evidence for change in level of labour input over time (Fig. 3d).

#### Soil disturbance levels

Figure 4a displays the disturbance model (Bogaard et al. 2022). Each smaller symbol represents a single modern arable plot (at either Laxton or Highgrove), coded according to the type of agricultural regime it represents and plotted along the discriminant function extracted to distinguish between high- and low-disturbance growing conditions (centroids are shown). Figure 4f displays the weed functional trait data used as discriminating variables and their relationship to the discriminant function. Figure 4b classifies the Sedgeford data based on the above model (Fig. 4a). Datapoints are clustered at the positive end of the axis, with discriminant analysis classifying all episodes as relatively high disturbance. Almost 90% of discriminant scores generated by our disturbance discriminant analysis exceed the ‘Lorsch baseline’, i.e. the lowest level of disturbance expected with use of a mouldboard plough (Fig. 4b).

According to Fig. 4d, there is little indication of a relationship between disturbance and area of the malting complex from which units derive. There is some suggestion (Fig. 4e) of a shift from a more to less soil-disturbed agricultural regime over time at Sedgeford, but the number of later-dated units is minimal.

### Stable isotope analysis

The δ^13^C_VPBD_ and δ^15^N_AIR_ values for each sample are presented in ESM 2. Associated %C, %N values and C:N ratios are also there detailed.

#### Carbon stable isotope values

**Fig. 5:**
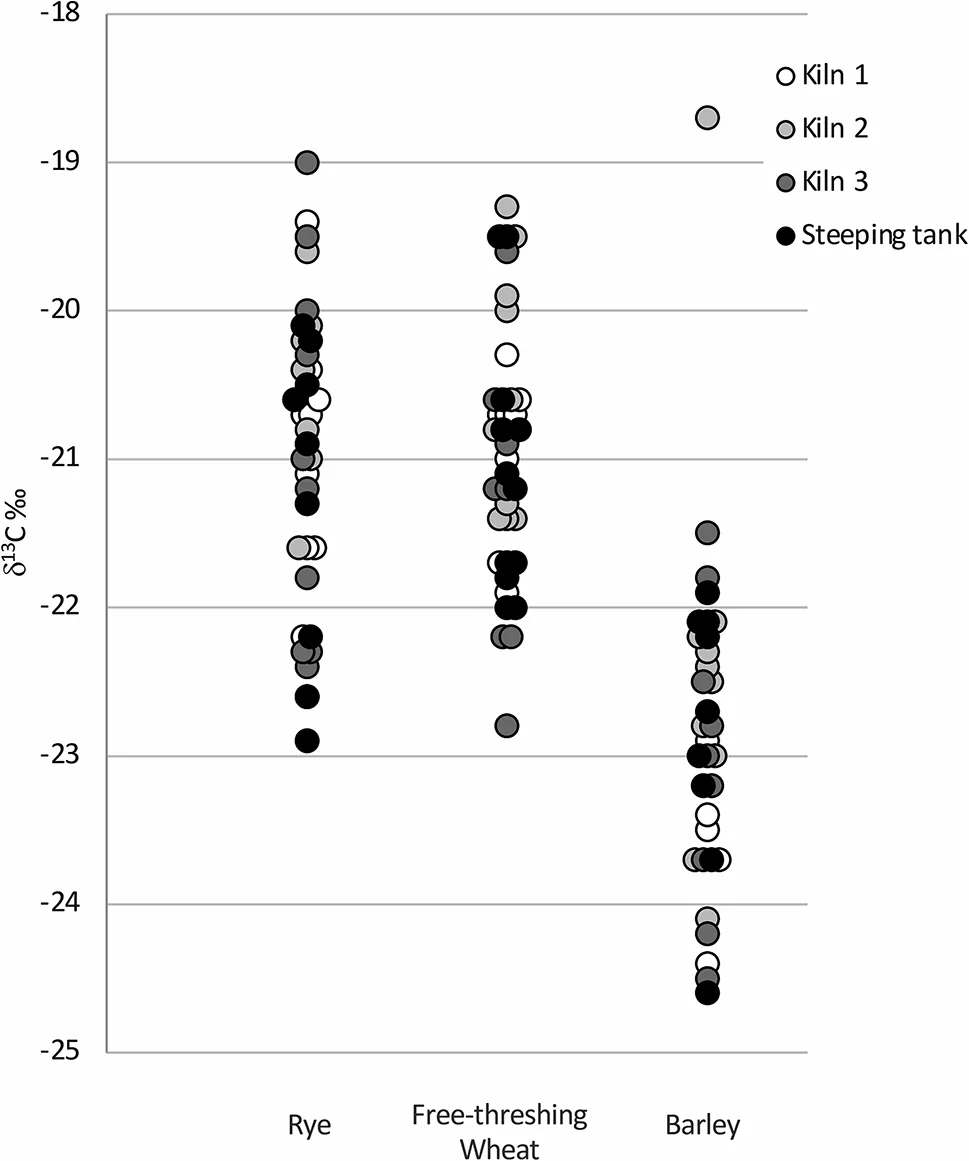
δ^13^C values for all samples, grouped by cereal taxon and coded by feature within the malting complex

**Fig. 6:**
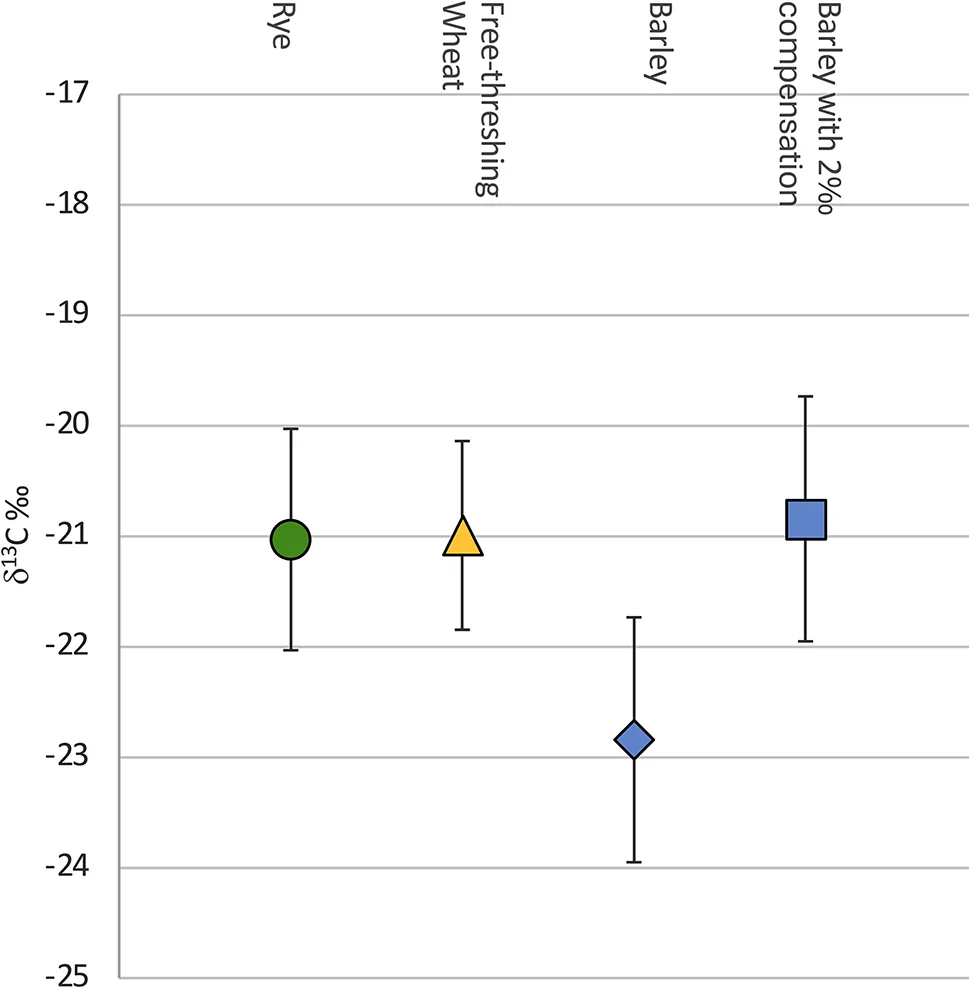
Mean δ^13^C values with associated SDs for all samples by cereal taxon. The mean for barley after compensating for expected 2‰ offset is shown

Figure 5 displays δ^13^C values for all samples, grouped by cereal taxon. The mean δ^13^C value for Sedgeford’s (six-row) barley of − 22.84 ± 1.11‰ is ~ 2‰ lower than that for both rye and wheat (Fig. 6; Table 4), a significant difference (F (2, 108) = 41.62, p = < 0.001) (ESM 1 Table S4). The expected − 2‰ offset in δ^13^C values for six-row barley if all taxa are cultivated in equal water conditions is thus observed. Adding 2‰ to the δ^13^C value for barley grains results in no significant difference between means (F (2, 108) = 0.20, *p* = 0.82) (ESM 1 Table S4).

**Table 4:** Number of samples, and mean δ^13^C values with SD for each cereal taxon analysed across all features, for all grains within each feature, and for each taxon by feature within the malting complex

Feature	Taxon	Number of (single grain) samples	Mean (‰)	Standard deviation (‰)
All	Rye	39	− 21.03	1.00
	Free threshing wheat	39	− 20.99	0.85
	Barley	33	− 22.84	1.11
Kiln 1^a^	All	23	− 21.07	0.69
Kiln 2		30	− 20.62	1.12
Kiln 3		29	− 21.08	1.04
Steeping tank		30	− 21.06	0.94
Kiln 1	Rye	9	− 20.92	0.82
	Free threshing wheat	9	− 20.94	0.52
	Barley	5	− 23.56	0.56
Kiln 2	Rye	10	− 20.93	1.01
	Free threshing wheat	10	− 20.56	0.84
	Barley	10	− 22.38	1.47
Kiln 3	Rye	10	− 20.97	1.23
	Free threshing wheat	10	− 21.24	0.94
	Barley	9	− 23.03	1.02
Steeping tank	Rye	10	− 21.19	1.03
	Free threshing wheat	10	− 21.12	0.96
	Barley	9	− 22.84	0.88

^a^ Considering the barley offset, single-grain samples from kiln 1, with only five barley grains, might be expected to have a lower mean *δ*^13^C value. However, ANOVA reveals no significant difference between mean *δ*^13^C values for each feature either with or without compensation for the barley offset. Mean values given here are those without compensation for an offset

δ^13^C values for rye and wheat have similar ranges (3.97‰ and 3.47‰ respectively) (Fig. 5). Excluding one outlier (a grain from context 19061: value − 18.66‰), the range for barley is 3.02‰. Stroud’s (2023) research found that the δ^13^C value SD for single grains from one modern field was ± 0.33‰, with a maximum range of 1.24‰: figures indicating expected variability in water availability within a single agricultural regime. SDs and ranges for all taxa exceed these thresholds, i.e. data are consistent with each crop deriving from a range of water availabilities (Figs. 5 and 6; Table 4). However, heterogeneity in other environmental conditions (e.g. soil type, canopy cover, light or topography) may also contribute to this variability.

Statistical comparison of crop means within each feature reveal, once 2‰ is added to δ^13^C values for barleys to compensate for the physiological difference, no significant disparities between taxa (ESM 1 Table S4). All SDs for grains of each cereal within each feature exceed the ± 0.33‰ threshold, i.e. variability in values for grains within each feature are consistent with cultivation in different water availability conditions (Nitsch et al. 2015; Stroud 2023).

**Fig. 7:**
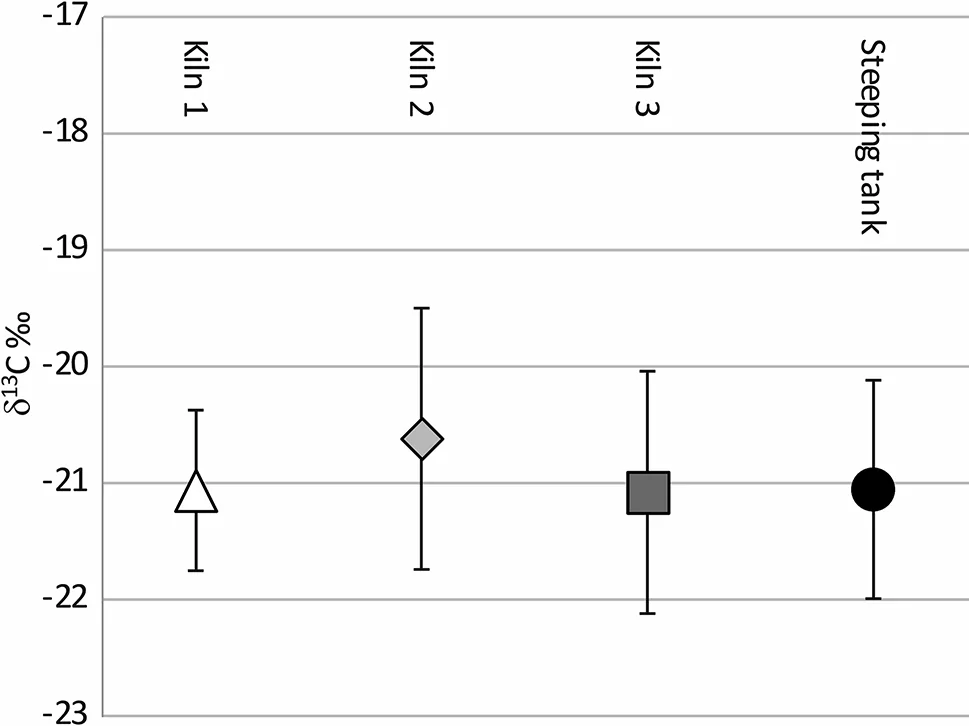
Mean δ^13^C values with associated SDs for all samples by feature within the malting complex. SDs are calculated after compensating 2‰ for the barley offset

No clear trends are apparent in the distribution of datapoints by feature within the malting complex (Fig. 5). This is also evident in Table 4 and Fig. 7, which show minimal difference in mean δ^13^C values between the features (where all taxa are combined), and this is confirmed statistically (F (3, 107) = 0.63, *p* = 0.60) (ESM 1 Table S5). After compensating for the barley offset, SDs for each feature exceed the ± 0.33‰ threshold, suggesting that grains between the features were cultivated in varying water conditions.

#### Nitrogen stable isotope values

**Fig. 8:**
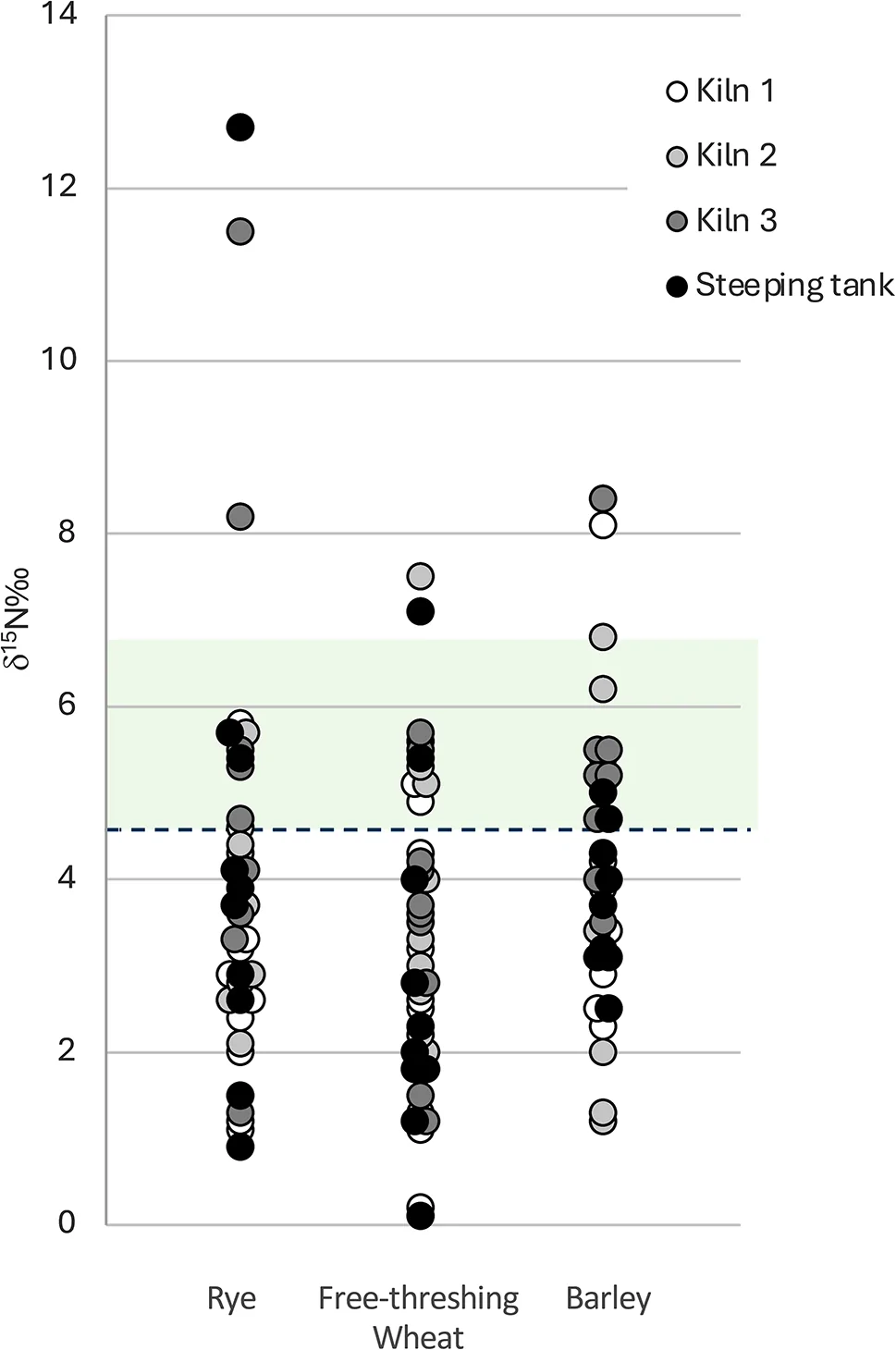
δ^15^N values for all samples, grouped by cereal taxon and coded by feature within the malting complex. The mean δ^15^N value for deer, minus 4‰ for the trophic shift (i.e. the wild herbivore baseline) is shown as a dashed line, with the expected grain/rachis offset shaded

Figure 8 displays δ^15^N values for all samples, grouped by cereal taxon. The data exhibit considerable variability, with δ^15^N values between 0.06 and 12.66‰; SDs are ± 2.46‰ (rye), ± 1.79‰ (wheat), and ± 2.07‰ (barley).

**Fig. 9:**
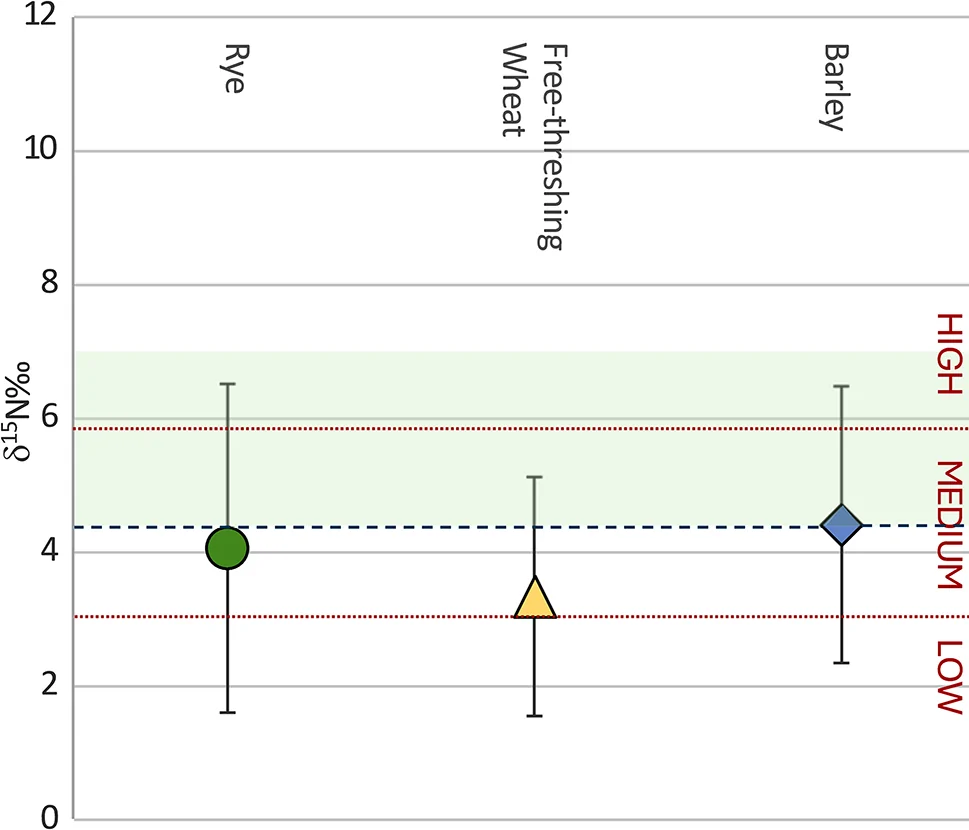
Mean δ^15^N values with associated SD for all samples by cereal taxon Bogaard et al. (2013) manuring bands (dotted lines) and the wild herbivore baseline (dashed) with grain/rachis offset shaded, are shown

Heterogeneity in manuring conditions can exist within a single field. However, modern crop studies using single grain samples suggest, for grains from a single arable experimental plot receiving ~ 25 tonnes of manure per hectare, a maximum expected range in δ^15^N of ~ 5.40‰, and maximum SD ~ ± 1.64‰ (Larsson et al. 2019). A comparable, unmanured plot had a maximum range of ~ 2.10‰, and maximum SD of ~ ± 0.56‰ (Larsson et al. 2019). These figures indicate the maximum expected variability within a single manuring regime. The implication is that each Sedgeford cereal taxon was cultivated under variable conditions in terms of manuring levels and/or in other environmental factors e.g. soil type, soil moisture level, or water table depth (cf. Hamerow et al. 2025, p 129). Most Sedgeford samples have low δ^15^N values compared to the calculated wild herbivore baseline (Fig. 8). 65% have values below the herbivore baseline. The low water content (21.07%) of Sedgeford’s free-draining soils (Sourced from the UK Soil Observatory CS Topsoil-Soil moisture map, https://mapapps2.bgs.ac.uk/ukso/home.html. Accessed 3 April 2025) may influence δ^15^N values; heavy, water-retaining soils are associated with increased δ^15^N values, likely due to the activity of denitrifying bacteria under anaerobic conditions (Hamerow et al. 2025, p 91). Figures 8 and 9 shows broad overlap in δ^15^N values for all taxa. Inter-crop differences are not statistically significant (F (2, 108) = 2.43, *p* = 0.09) (ESM 1 Table S6). Means for both rye and free-threshing wheat are both less than the wild herbivore baseline, with barley only slightly above (Fig. 9; Table 5). The averaged (‘bulked’) single grain values in Fig. 9 may viably be compared with Bogaard et al.’s (2013) manuring bands for expected δ^15^N values based on bulk samples of modern grains from plants experimentally cultivated in differing manuring regimes. Figure 9 shows that the wild herbivore baseline calculated for Sedgeford falls in the middle of the ‘medium’ manuring band as calculated by Bogaard et al.: clearly, these bands assume a different (lower) baseline value, and hence some caution is required in their interpretation with reference to Sedgeford data. Mean values for each Sedgeford crop taxon fall within the lower part of the ‘medium’ Bogaard et al. manuring band (2013, Fig. 1).

**Table 5:** Number of samples, and mean δ^15^N values with associated SD, for each cereal taxon across all features, for all grains within each feature, and for each taxon by feature within the malting complex

Feature	Taxon	Number of (single grain) samples	Mean δ^15^*N* (‰)	Standard deviation (‰)
All	Rye	39	4.06	2.46
	Free threshing wheat	39	3.34	1.79
	Barley	33	4.41	2.07
Kiln 1	All	23	3.09	1.78
Kiln 2		30	4.16	2.08
Kiln 3		29	4.59	2.13
Steeping tank		29	3.63	2.35
Kiln 1	Rye	9	2.96	1.53
	Free threshing wheat	9	2.80	1.72
	Barley	5	3.84	2.41
Kiln 2	Rye	10	3.84	1.23
	Free threshing wheat	10	4.08	1.76
	Barley	10	4.56	3.01
Kiln 3	Rye	10	5.01	2.94
	Free threshing wheat	10	3.58	1.45
	Barley	9	5.23	1.36
Steeping tank	Rye	10	4.32	3.30
	Free threshing wheat	10	2.83	1.97
	Barley	9	3.75	0.82

Statistical comparison of mean δ^15^N values for crop taxa within each feature also suggests no significant differences (ESM 1 Table S6). Table 5 shows SDs for each taxon within each feature. Most sets of grain taxa within each feature (8/12) have a SD > ± 1.64‰ (Larsson et al.’s (2019) threshold for heavily manured fields), suggesting origins in more than one condition of manuring.

Significantly, SDs for each grain taxon within each feature (the mean for these being ± 1.96‰, *n* = 12) suggest almost as much variability in grain δ^15^N values as equivalent SDs for each crop across all features (mean for these SDs being ± 2.10‰, *n* = 3) (Table 5). The likely chronological separation of kiln 3 (later) from kilns 1 and 2 has been observed. Grains from different features were probably distinct not only in date but also in field-of-origin. Evidence for comparable variability within and between features implies significant heterogeneity in cultivation conditions within each field supplying the malting complex.

**Fig. 10:**
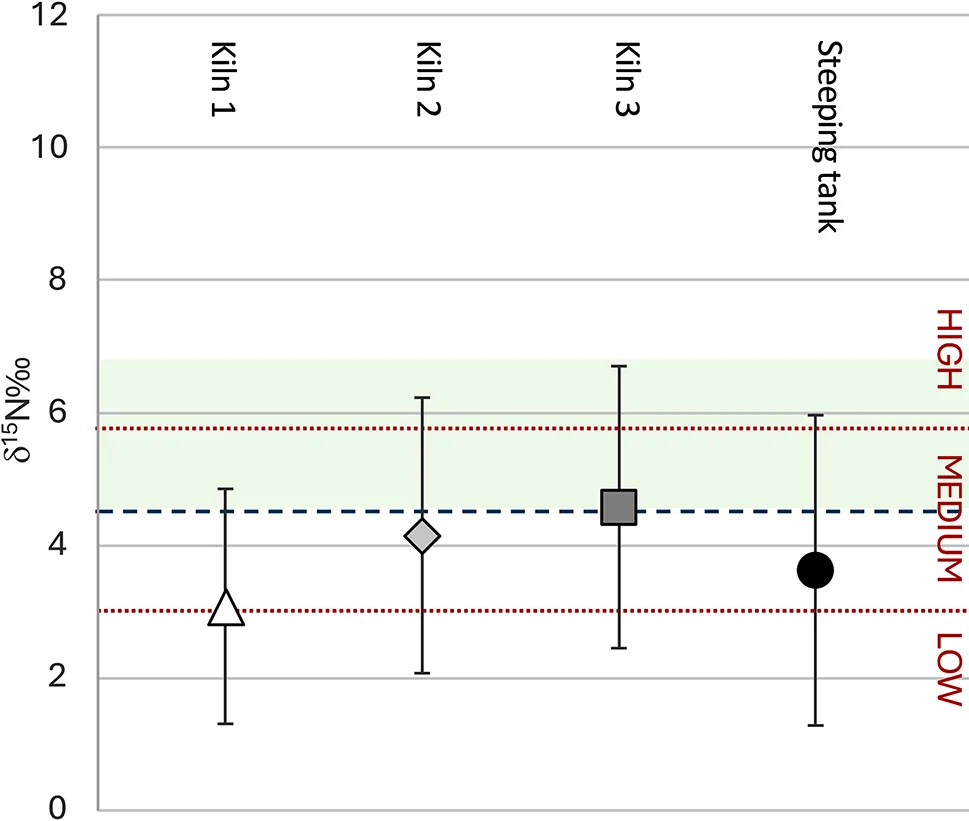
Mean δ^15^N values with associated SD for all samples by feature within the malting complex. Manuring bands (dotted lines) and the wild herbivore baseline (dashed line) are shown, with the grain/rachis offset shaded

No trends are evident in the distribution of samples by feature type (Fig. 8). Differences in mean δ^15^N values between the features are not significant (F (3, 107) = 2.47, *p* = 0.07) (Fig. 10; Table 5; ESM 1 Table S7). SDs for samples (of all taxa) grouped by feature all exceed the ± 1.64‰ upper limit for grains from a single source (Table 5), implying grains recovered across features grew in more than one manuring condition.^2021^

One might imagine, being close to the coast, that seaweed (macroalgae) or fish remains were used to fertilise crops malted at Sedgeford. This is unlikely. Modern research (Gröcke et al 2021) suggests use of both seaweed and fish remains as fertiliser i.e. ‘marine biofertilisation’ cause elevated nitrogen stable isotopic values in soils and associated crop plants. The low stable nitrogen isotope values at Sedgeford suggest marine biofertilisation was not in use.

#### Comparing carbon and nitrogen stable isotope values

**Fig. 11:**
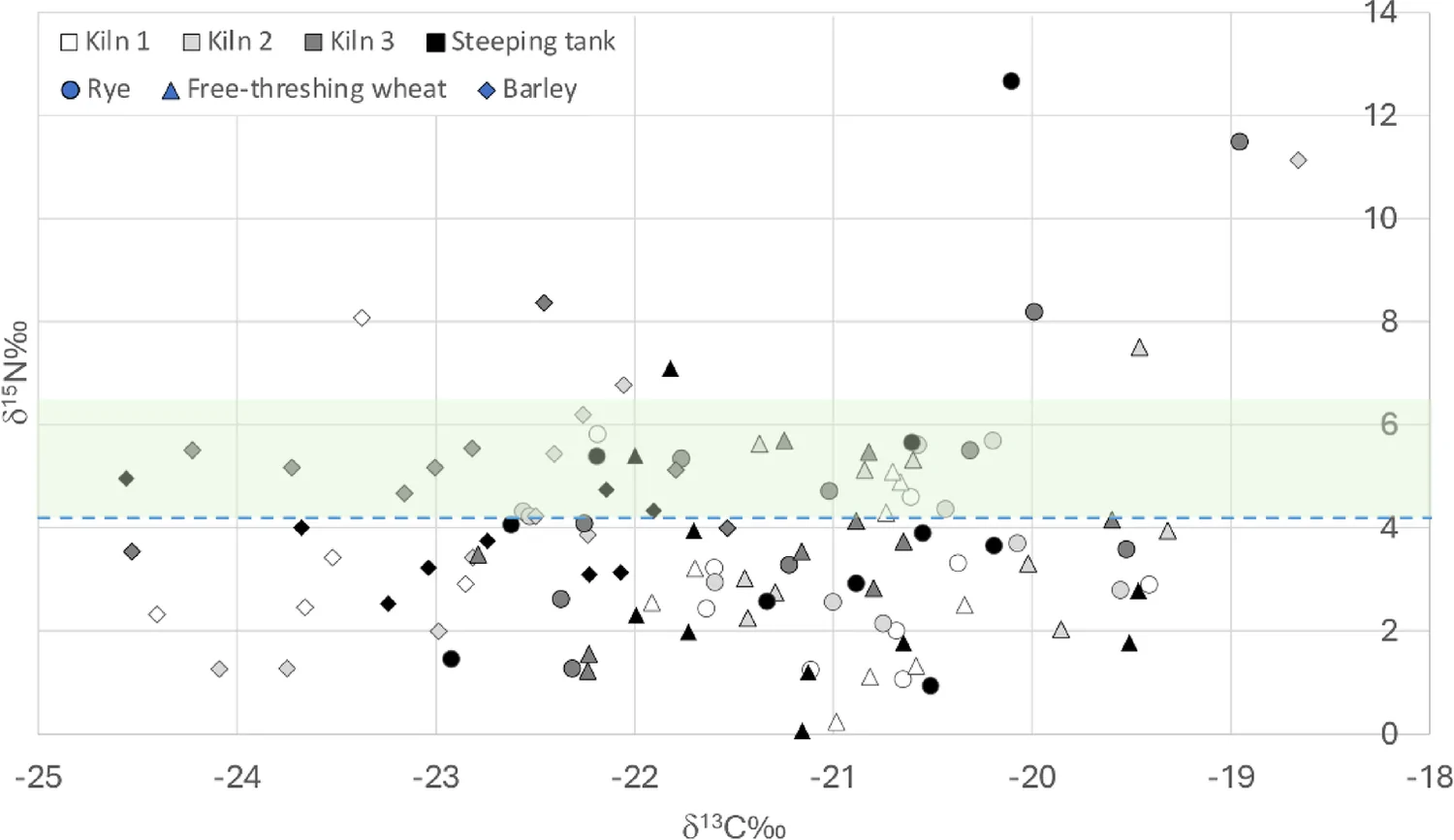
δ^15^N values plotted against δ^13^C values for all samples, coded by cereal taxon and feature within the malting complex. The wild herbivore baseline is shown as a dashed line and expected grain/rachis offset shaded

Figure 11 displays δ^15^N values plotted against δ^13^C values for all grains, coded by taxon and area of the malting complex. Datapoints of each type show considerable overlap and there is no significant relationship between δ^15^N and δ^13^C values (Pearson’s product-moment correlation co-efficient = 0.15, p-value = 0.12).

Five clear outlier datapoints (representing different taxa and malting complex features) have elevated values for both δ^13^C (>–21‰) and δ^15^N (> 7‰), seemingly attributable neither to measurement or machine error, nor to post-depositional contamination. These may relate to salinity (Sedgeford approximating the coastal zone); research suggests that salinity may elevate both plant δ^15^N and δ^13^C values (Heaton 1987; van Groenigen and van Kessel 2002; Yousfi et al. 2010; Hussain and Al-Dakheel 2018). Elevated δ^13^C values in saline environments likely relate to partial stomatal closure in plants under salt stress, whilst elevated δ^15^N may be due to raised pH acting to increase discriminating volatisation of lighter N^14^ isotopes in soil (van Groenigen and van Kessel 2002).

It has been argued (e.g. Caroe 2022b, p 340; Faulkner 2022) that Sedgeford is an example of an early medieval ecclesiastical or lordly estate centre. Supportive evidence for Sedgeford’s representing an estate centre under ecclesiastical control includes the recovery of two metal writing styli from the cemetery part of the site (Jolleys et al. 2019, p 76). Styli at other early medieval sites, including Flixborough, have been associated with elite ecclesiastical oversight, although this is somewhat contentious (Loveluck 2001). Were this the case, it is to be expected that the site would receive imported crops cultivated on an agricultural ‘hinterland’, extending some distance away, for processing in the malting complex: an alternative possible explanation for outlier δ^13^C and δ^15^N values.

### Seasonality

**Fig. 12:**
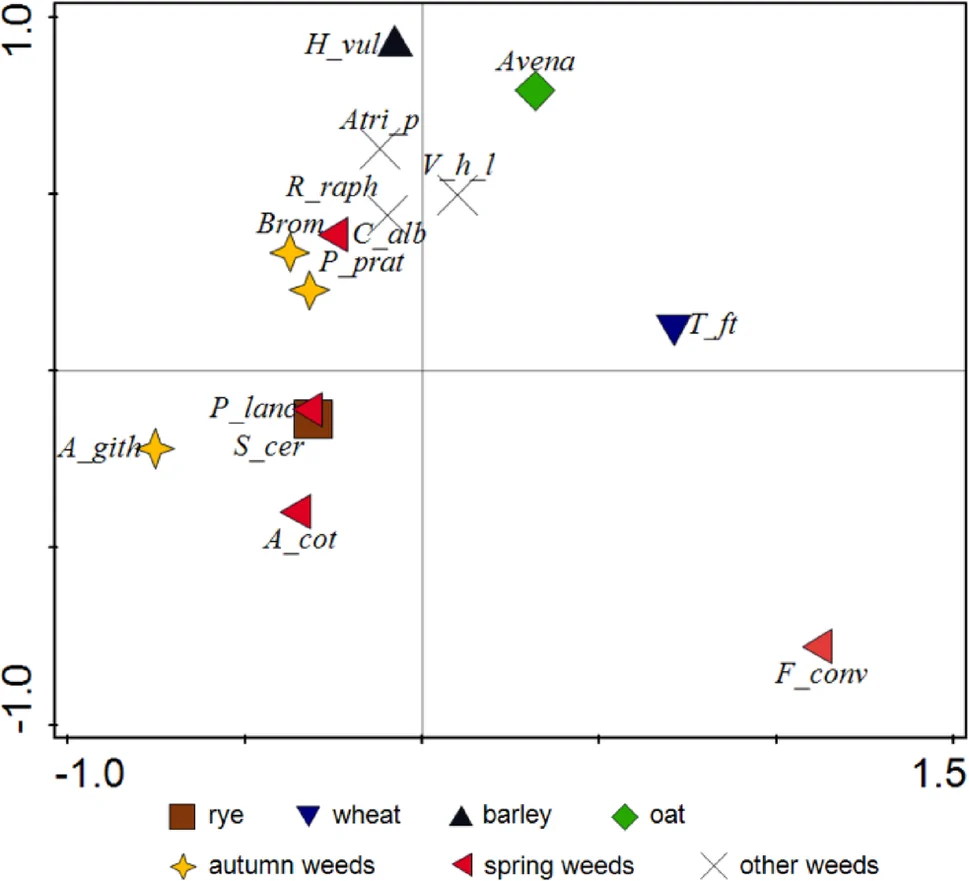
Correspondence analysis plot showing cereal taxa and weed species distributed according to associations in samples from the assemblage. Weeds are coded according to seasonality class

**Fig. 13:**
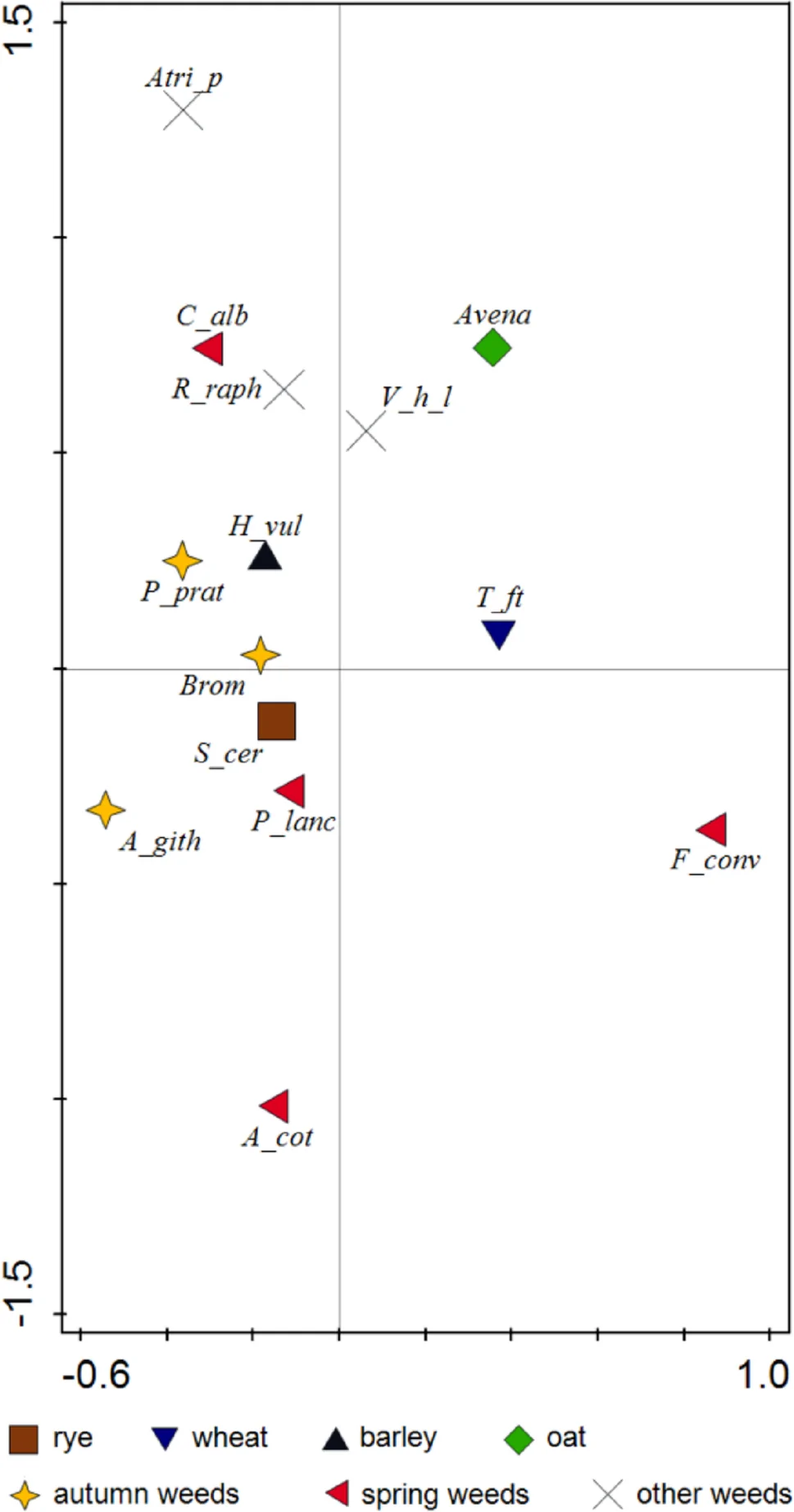
Correspondence analysis as in Fig. 12, with square root transformation applied to data

‘Seasonality’ scatterplots – the output of correspondence analysis – are displayed in Figs. 12 and 13. In each case, weed species are coded by sowing time association (Table 2). Figure 13 shows the same data following square root transformation. Seven weed species (out of 11 eligible) from the assemblage are clearly associated with a sowing season (i.e. are either autumn or spring-germinating).

All three autumn-germinating weed taxa strongly cluster together towards the negative (left) end of the x-axis together with rye. In Fig. 13, this cluster also includes barley. Wheat occurs at the positive (right) end of the x-axis, the only nearby seasonality-associated weed being (spring-germinating) *Fallopia convolvulus*. Oat also falls towards the positive end of the x-axis. Of the most abundant weed species in the assemblage (Table 3), two are clearly associated with a particular crop taxon: spring-germinating *Fallopia convolvulus* with wheat and autumn-germinating *Agrostemma githago* with rye.

Occurrence in both plots of (spring-germinating) *Plantago lanceolata*, *Anthemis cotula* and *Chenopodium album* in a cluster with autumn-sown rye and all three autumn-associated species at the negative end of the x-axis likely reflects the recognised capacity for spring-germinating species to persist among autumn-sown crops (Bogaard et al. 2001). Some mixing is expected among autumn-sown crops since autumn ploughing advantages autumn-germinating weeds but does not exclude spring-germinating taxa (by contrast, spring sowing destroys autumn-germinating weeds). This means that autumn-germinating weeds are a stronger indicator of autumn sowing than spring-germinating are of spring sowing.

To summarise, the correspondence analysis scatterplots are compatible with consistent and complementary sowing seasons for different cereals and by implication, crop rotation. The implication is that rye was an autumn-sown crop, with barley perhaps also autumn-sown. Wheat and oat were probably spring-sown. Given that rye dominates the assemblage, it is not surprising that, of the four cereals, rye shows the clearest seasonality pattern.

## Discussion

### The ‘mouldboard plough package’ at Sedgeford?

Results presented above are here synthesised and contextualised as part of the story of agriculture in 8th and 9th c. ad England. We consider each element of the mouldboard plough package in turn.

#### Extensification

As noted above, across England and beyond, there was a marked tendency in the 7th to 9th c. ad for farmers to expand cereal production. The FWE ‘intensity’ model reveals that crops from the Sedgeford malting complex were cultivated extensively. This accords with evidence from isotopic analysis for low δ^15^N values, indicating crops were grown in low manuring regimes. As noted above, both the wild herbivore baseline and Bogaard et al. (2013) manuring ‘bands’ used here as reference points to assess the level of soil manuring indicated by results from stable nitrogen isotope analyses must, for differing reasons, be treated with some caution. However, the low soil fertility indicated by the evidence from FWE supports our conclusion that Sedgeford’s was a low manuring regime.

#### Mouldboard plough

The disturbance discriminant analysis suggests high levels of disturbance at Sedgeford, consistent with use of a mouldboard plough (Hamerow et al. 2020; Bogaard et al. 2022). Mouldboard ploughing of the soil around Sedgeford (bringing nutrients for plant growth to the surface) may have been a strategy used by local farmers to compensate for the soil’s low fertility. Faulkner (2022) presents additional evidence for mouldboard plough use at Sedgeford, including plough marks on malting complex features. By the ad 1086 Domesday survey, the settlement at Sedgeford is associated with five plough teams (Faulkner 2022). Evidence for high levels of disturbance, consistent with mouldboard plough use, at 8th and 9th c. ad Sedgeford reflects a trend revealed at several early medieval sites, including Stafford (West Midlands) and Lyminge (Kent) (Bogaard et al. 2022).

#### Crop rotation

Evidence in an archaeobotanical assemblage for different crops being cultivated in similar environmental conditions is consistent with crop rotation (Hamerow et al. 2025, pp 104–138). Hamerow et al. (2025, pp 106–108) have outlined a set of criteria for distinguishing between samples comprising crops cultivated separately (as a single, unmixed crop without rotation), together as a mixed crop (such as a maslin) without rotation, or in (two or three-course) rotation, respectively (Table 6). We address these in turn.

**Table 6:** (a) Criteria for determining whether remains of different crops in archaeobotanical samples were cultivated as a mixed crop, in rotation or separately, after Hamerow et al. (2025 Table 4.1 p.107); (b) An assessment of how crops in samples from the Sedgeford assemblage meet these criteria

Category	Mixed crop	2-course rotation	3-course rotation	Separate cultivation
(a)				
Crop carbon and nitrogen stable isotope values	Compatible	Compatible	Compatible	Incompatible
Disturbance levels	Unclear	High	High	Unclear
Crop sowing times	Compatible	Compatible or contrasting	Contrasting	Compatible or contrasting
Cereal composition	Mixed	Mixed or pure	Mixed or pure	Pure
(b)				
Crop carbon and nitrogen stable isotope values	✓	✓	✓	×
Disturbance levels	?	✓	✓	?
Crop sowing times	×	✓	✓	✓
Cereal composition	✓	✓	✓	×

First, results reveal that malting complex crops have compatible carbon and nitrogen stable isotope values (i.e. similar mean values for carbon, and for nitrogen, respectively, both within and across all features). This is consistent with mixed cropping, two-course or three-course crop rotation but excludes separate cultivation. Secondly, disturbance levels for Sedgeford’s crops are high; two- or three-course rotation is particularly associated with high disturbance. Evidence for crop sowing times as revealed through correspondence analysis suggests autumn *or* spring sowing of particular crops, consistent with crop rotation. Finally, crop deposits from across the malting complex are consistently mixed. It is possible that grains of different crops were mixed post-harvest, purposely for malting. This would not affect our conclusions concerning the cultivation of crops recovered at Sedgeford either separately, as a mixed crop or in rotation (Table 6b). In sum: the Sedgeford material is consistent with a dominant farming regime in fields supplying the malting complex of two- or three-course crop rotation, but not with mixed cropping or separate crop cultivation. Clear associations of particular weed seeds with certain cereal taxa in the assemblage (*Fallopia convolvulus* with wheat, and *Agrostemma githago* with rye) are also both consistent with crop rotation and inconsistent with the ‘maslin hypothesis’ (mixed cropping).

Variability in δ^15^N values implies that grains were cultivated in heterogeneous conditions – in several fields, a single field in successive seasons or a single field with heterogenous conditions. One type of crop husbandry system involving ‘single fields with heterogenous conditions’ in use at Sedgeford could have been *open field* farming, with crops cultivated in ‘strips’ (*selions*) differentially manured by different farmers, and using a system of crop rotation. However, evidence for ‘classic’ open field farming systems generally post-dates the 7th to 9th c. ad, and systems are concentrated in England’s ‘Central Province’. Classic open field farming is thus an unlikely explanation for isotopic trends at 8th and 9th c. ad, East Anglian, Sedgeford (Thirsk 1964, p 23; Hall 2014, pp 2–3; Williamson 2022).

If systematic crop rotation (though likely not an ‘open field’ system) were occurring at 8th and 9th c. ad Sedgeford, this would be remarkably early, though perhaps not unprecedented. There is suggestion of patterning in crop sowing times in correspondence analyses for the assemblage from West Fen Road, Ely (Cambs), dated ca. ad 720–1220, whilst isotopic results from Mildenhall (Suffolk) and Holmer (Herefordshire), both dated ca. ad 770–880, are compatible with systematic rotation (Hamerow et al. 2025, pp 118–119, 131).

### Sedgeford’s place in the long agricultural ‘revolution’

Archaeologists and historians have long been eager to identify ‘revolutionary’ change within their own period of interest (Williamson 2022). FeedSax research suggests that, while the 7th to 9th c. ad saw significant innovations, no single period in medieval England witnessed ‘revolutionary’ agricultural transformation (Hamerow 2022): if the medieval era saw a revolution in English agriculture, it was a ‘long’ one (Hamerow et al. 2025). Not until the 10th to 11th c. ad is there persuasive evidence for widespread systematic crop rotation and mouldboard plough use, although these are earlier evidenced at some sites from the 8th c. ad onwards (Hamerow 2022).

The FeedSax three-fold ‘mouldboard plough package’ is a helpful framework for considering changes, or lack thereof, in medieval agriculture (McKerracher and Hamerow 2022). We have found at least tentative evidence in the malting complex assemblage for each element of this ‘package’ in fields supplying 8th and 9th c. ad Sedgeford.

The presence of a multi-kiln malting complex at Sedgeford is part of the emerging picture of renewed construction of specialised grain processing (e.g. watermills, corn-dryers and malting kilns) and storage features from the 7th to 9th c. ad in England, for the first time since the Romano–British era (Hamerow 2012, pp 151–152). Further, the richness of crop remains from the malting complex is consistent with a quantitative trend to sites with rich crop assemblages from the 7th c. (McKerracher 2016, 2018, 2019). Each trend implies increased arable production.

The assemblage moreover exhibits crop diversification; this goes hand in hand with growth in arable surpluses through careful matching of crop choices to local environmental conditions (McKerracher 2018, p 120). The unusual abundance of rye in the malting complex suggests such ‘careful matching’: rye was generally a minor crop in Anglo-Saxon England (Hagen 2006, p 38; Comeau and Burrow 2021), but there are indications of geographic foci for rye cultivation in parts of East Anglia in the 7th to 9th c. ad in regions of well-drained soils, to which rye is well adapted (Murphy 1985; Rippon et al. 2015, p 172; McKerracher 2018, p 105). For other potential factors influencing the selection of rye as the malting complex’s major crop, see Caroe (2022a). Our research suggests that, if an ‘agricultural revolution’ can in any way be claimed to have taken place from the 7th c. ad in England, Sedgeford was a part of it.

## Conclusions

Beer to drink was just as important as bread to eat, both practically and symbolically, for the peoples of 7th to 9th c. ad England. Yet corresponding archaeobotanical evidence has been, to date, conspicuously lacking – hence the particular significance of an assemblage from the earliest unambiguous multi-feature Anglo-Saxon malting complex, revealed at Sedgeford. Archaeobotanical research, uncovering an abundance of germinated grains (a sign of malting), was the key ‘missing piece’, alongside structural evidence, underpinning the designation of Sedgeford’s trench 23 as an 8th and 9th c. ad malting complex.

FWE methods, stable isotopic analyses and weed seasonality analyses have revealed evidence for all three components of the so-called ‘mouldboard plough package’ – extensification of cultivation, use of a mouldboard plough, and perhaps, early crop rotation – in the fields that supplied Sedgeford’s malting complex. Additional characteristics of the malting complex assemblage exemplify other trends associated with the period. These include its sheer richness – evidencing local specialisation (in grain processing for beer-production), and also a diverse crop spectrum – dominated by rye, a minor crop in Anglo-Saxon England but well adapted to local environmental conditions. This was a time when farmers were ‘digging themselves in as never before’ (McKerracher 2018, p 118) – investing in their connection with the land – to both feed and slake the thirst of a people who esteemed the ploughman as one giving ‘drink as well as bread’ (Ælfric’s Colloquy, Garmonsway 1991, p 40, l.226).

## Supplementary Information

Below is the link to the electronic supplementary material.

Supplementary Material 1

Supplementary Material 2

## Abbreviations

FWE: functional weed ecology

## Declarations

The research presented here was conducted as part of Hannah Caroe’s doctoral studies at the School of Archaeology, University of Oxford, co-supervised by Amy Bogaard and Michael Charles. Hannah Caroe and Amy Bogaard contributed to the study conception and design. Material preparation, data collection and analysis were performed by all authors. The first draft of the manuscript was written by Hannah Caroe and all authors commented on previous versions of the manuscript. All authors read and approved the final manuscript.
