# Supplementary material for: Archaeobotanical evidence reveals the nature of cereal agriculture at 8th- and 9th-century ad Sedgeford, East Anglia, UK
Source: Veg Hist Archaeobot. 2026 May 19;35(5):719–37. doi: 10.1007/s00334-026-01091-w (PMC13499822; doi:10.1007/s00334-026-01091-w)
Supplement: Supplementary file 1 [file 334_2026_1091_MOESM1_ESM.pdf]

Supplementary Material 1

Article title: Archaeobotanical evidence reveals the nature of cereal agriculture at eighth- and ninth-century A.D. Sedgeford, East Anglia, U.K.

Journal: Vegetation History and Archaeobotany

Hannah Caroe[1], Amy Bogaard and Elizabeth Stroud

1 = corresponding author

University of Oxford, Hannah.caroe@arch.ox.ac.uk

Table S1 *Characterising nitrogen stable isotope values for two wild herbivore individuals from Anglo-Saxon East Anglia*

Descriptive category	Wild herbivore 1	Wild herbivore 2
**Reference**	Leggett et al. 2021	Purcell 2012
**Location**	Stanton, W. Suffolk	Lakenheath, Suffolk
**Distance from Sedgeford**	41.9 miles	34.6 miles
**Soil moisture**	22%	30%
**Date**	5th to 9th centuries	5th to 7th centuries
**Species**	Red Deer	‘deer’ (species not recorded)
**Bone type**	Astragalus	Tibia
**δ^{15}N value**	6.1‰	5.7‰
**δ^{15}N value after compensating for offsets** (expected value for unmanured grains)	4.5‰	4.1‰
**Mean expected δ^{15}N value for unmanured grains**	4.3‰	

Fig. S1 *ATR-FTIR plots comparing spectra for artificially contaminated samples with archaeological grains from Sedgeford. Plots shown examine potential contamination with a) carbonates, b) nitrates and c) humic acids. In each case, lower spectra are from Vaiglova* et al. *(2014) and the three uppermost spectra from Sedgeford grains*

a)

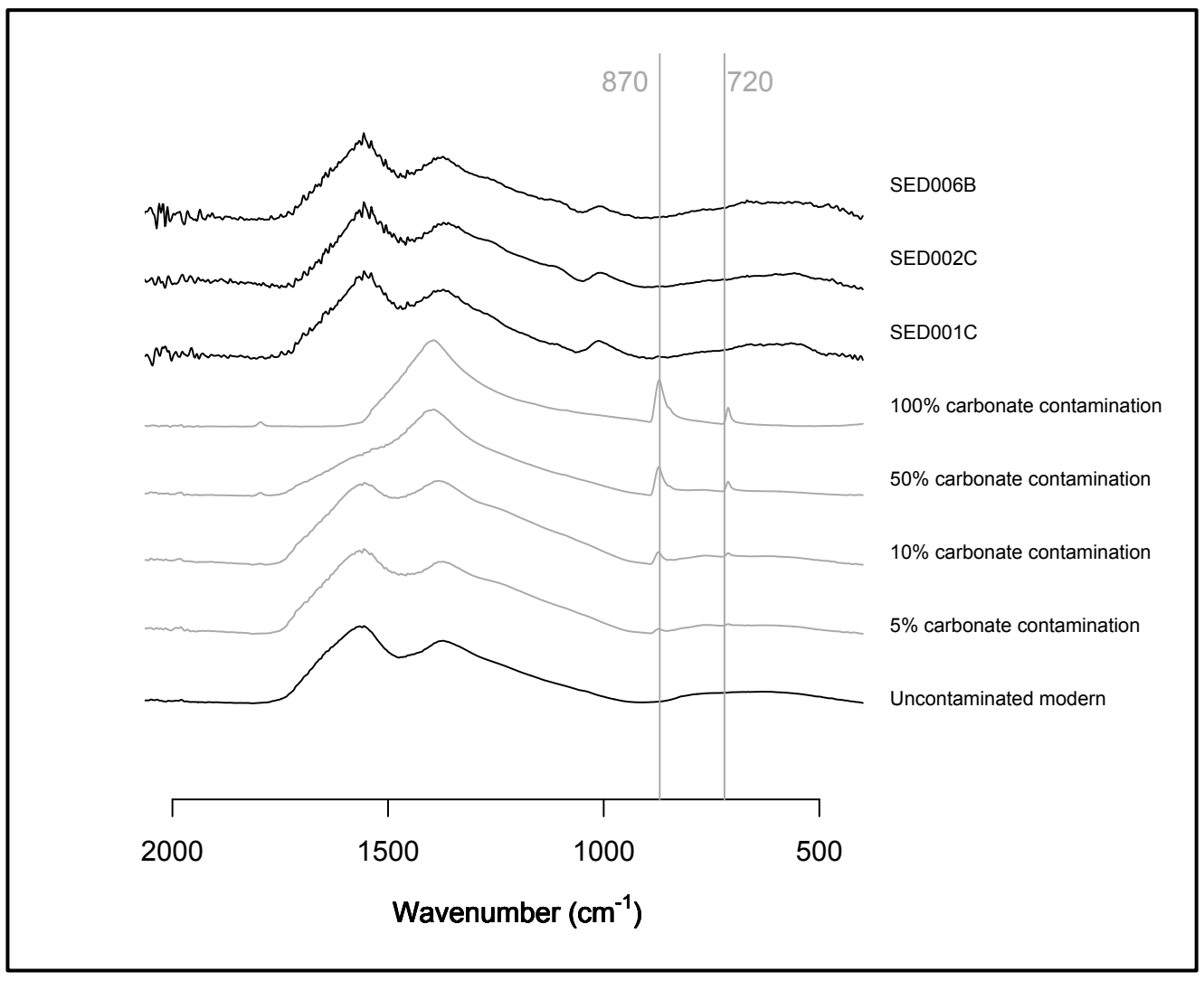

b)

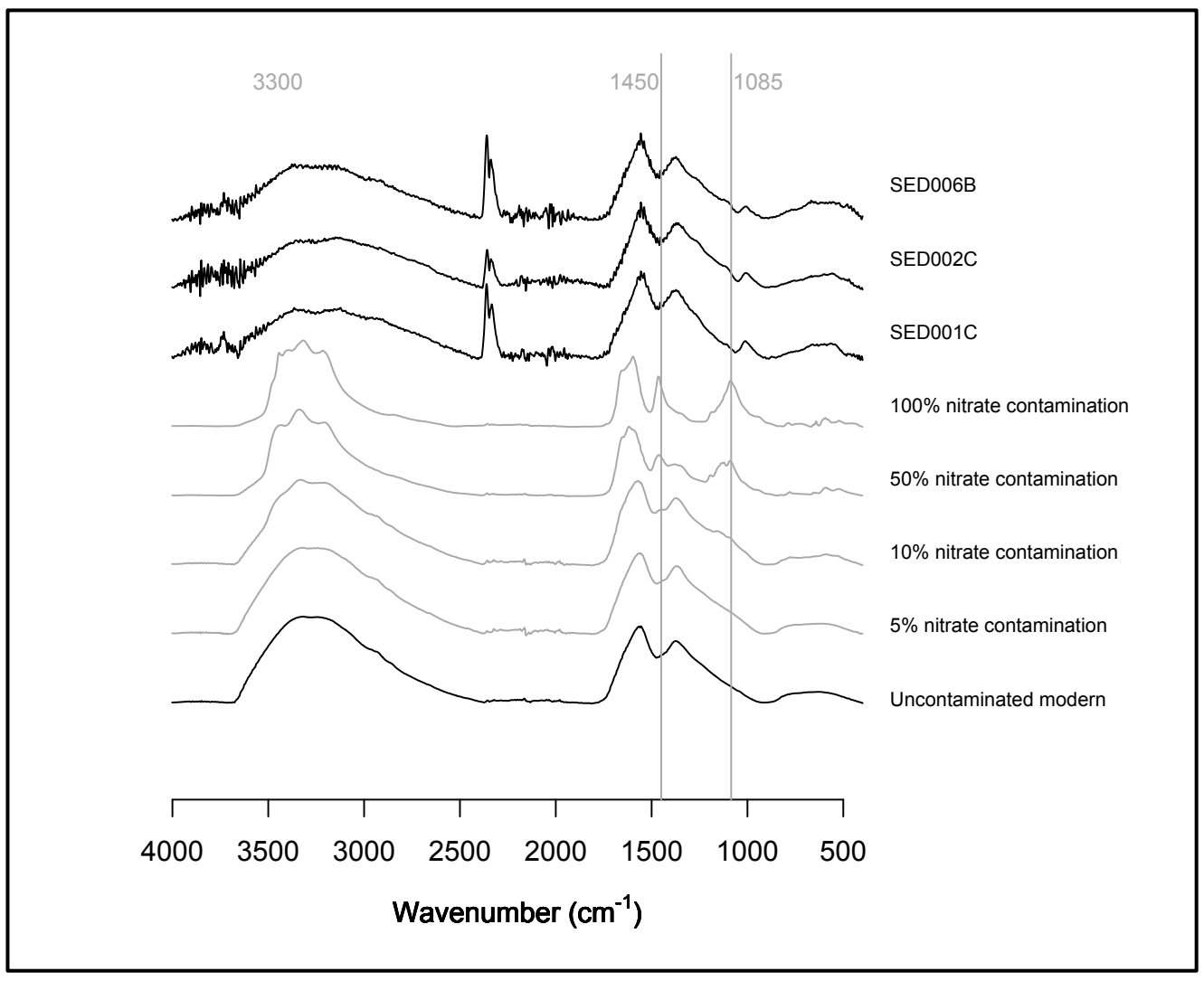

c)

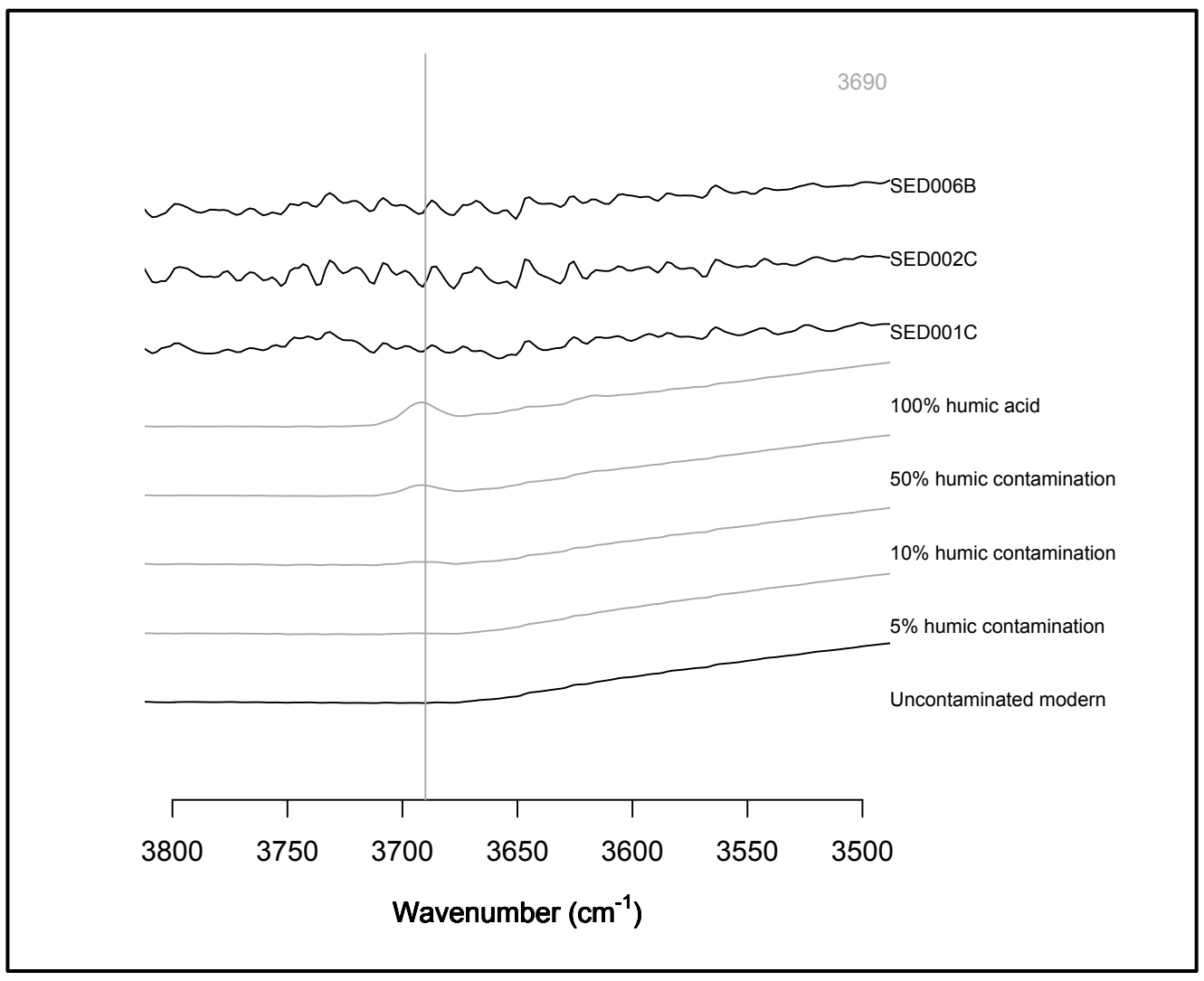
3690
SED006B
SED002C
SED001C
100% humic acid
50% humic contamination
10% humic contamination
5% humic contamination
Uncontaminated modern
3800
3750
3700
3650
3600
3550
3500
Wavenumber (cm-1)

Table S2 *Summarising frequency of single-grain samples selected for stable isotope analysis, per species, context and feature type, with allocated isotope analysis code*

Feature	Context number	Species	Number of samples	Isotope analysis code
**Kiln 1**	23719	rye	5	SED01
		FT wheat	4	SED02
		hulled barley	0	
	23754	rye	4	SED04
		FT wheat	5	SED05
		hulled barley	5	SED06
**Kiln 2**	23722	rye	5	SED07
		FT wheat	5	SED08
		hulled barley	5	SED09
	19061	rye	5	SED10
		FT wheat	5	SED11
		hulled barley	5	SED12
**Kiln 3**	23375	rye	5	SED16
		FT wheat	5	SED17
		hulled barley	5	SED18
	23646 G/H7	rye	5	SED19
		FT wheat	5	SED20
		hulled barley	4	SED21
**Steeping tank**	19049	rye	5	SED28
		FT wheat	5	SED29
		hulled barley	4	SED30
	23621	rye	5	SED31
		FT wheat	5	SED32
		hulled barley	5	SED33
**TOTAL**	111			

Table S3 *Means and standard deviations for calibration and check standards used in mass spectrometer runs for Sedgeford samples*

Standard	Type	$\delta^{13}C$ ‰		$\delta^{15}N$ ‰	
		Mean	Standard deviation	Mean	Standard deviation
Alanine	Calibration	-26.91	±0.11	-1.57	±0.13
Cow	Calibration	-24.28	±0.12	7.86	±0.18
Seal	Calibration	-12.54	±0.15	16.14	±0.24
EMA-P2	Check	-28.19	±0.16	-1.57	±0.29
Leucine	Check	-28.24	±0.21	6.29	±0.24

Table S4 *ANOVA and Tukey post-hoc results for differences in mean $\delta^{13}C$ values between cereal taxa, across all features and within features from the malting complex. All tests were performed first without and secondly with compensation for the 2‰ barley offset. P-values indicating significant differences at the 0.05 level are marked with an asterisk (*)*

Feature	Barley offset	ANOVA			Tukey post-hoc test
		F-ratio	Degrees of freedom	P-value	
All	No compensation	41.62	2, 108	<0.001*	rye-wheat. 0.98<br>wheat-barley <0.001*<br>rye-barley <0.001*
	2‰ compensation	0.2	2, 108	0.82	
Kiln 1	No compensation	30.56	2, 20	<0.001*	rye-wheat 0.10<br>wheat-barley <0.001*<br>rye-barley <0.001*
	2‰ compensation	1.77	2, 20	0.20	
Kiln 2	No compensation	7.1	2, 27	0.03*	rye-wheat 0.75<br>wheat-barley 0.004*<br>rye-barley 0.02*
	2‰ compensation	0.61	2, 27	0.55	
Kiln 3	No compensation	10.1	2, 26	<0.001*	rye-wheat 0.84<br>wheat-barley 0.003*<br>rye-barley. <0.001*
	2‰ compensation	0.17	2, 26	0.84	
Steeping tank	No compensation	9.62	2, 26	<0.001*	rye-wheat. 0.98<br>wheat-barley 0.02*<br>rye-barley 0.03*
	2‰ compensation	0.34	2, 26	0.71	

Table S5 *ANOVA results for differences in mean $\delta^{13}C$ values between features within the malting complex for all cereal taxa and for each taxon, respectively*

Taxon	ANOVA		
	F-ratio	Degrees of freedom	P-value
**All**	0.63	3, 107	0.6
**Rye**	0.15	3, 35	0.92
**Wheat**	1.23	3, 35	0.31
**Barley**	1.39	3, 29	0.27

Table S6 *ANOVA results for differences in mean $\delta^{15}N$ values between cereal taxa, across all features and within features from the malting complex*

Feature	ANOVA		
	F-ratio	Degrees of freedom	P-value
**All**	2.43	2, 108	0.09
**Kiln 1**	0.56	2, 20	0.58
**Kiln 2**	0.29	2, 27	0.75
**Kiln 3**	1.82	2, 26	0.18
**Steeping tank**	1.03	2, 26	0.37

Table S7 *ANOVA results for differences in mean $\delta^{15}N$ values between features, across all taxa and for each cereal taxon*

Taxon	ANOVA		
	F-ratio	Degrees of freedom	P-value
**All**	2.47	3, 107	0.07
**Rye**	1.19	3, 35	0.33
**Wheat**	1.20	3, 35	0.32
**Barley**	0.92	3, 29	0.45
