# Supplementary material for: Archaeobotanical evidence reveals the nature of cereal agriculture at 8th- and 9th-century ad Sedgeford, East Anglia, UK
Source: Veg Hist Archaeobot. 2026 May 19;35(5):719–37. doi: 10.1007/s00334-026-01091-w (PMC13499822; doi:10.1007/s00334-026-01091-w)
Supplement: Supplementary file 2 [file 334_2026_1091_MOESM2_ESM.pdf]

Supplementary Material 2: Stable isotope data

Article title: Archaeobotanical evidence reveals the nature of cereal agriculture at eighth- and ninth-century A.D. Sedgeford, East Anglia, U.K.

Journal: Vegetation History and Archaeobotany

Hannah Caroe[1], Amy Bogaard and Elizabeth Stroud

1 = corresponding author

University of Oxford, Hannah.caroe@arch.ox.ac.uk

Carbon and nitrogen stable isotope data for single-grain samples from the Sedgeford malting complex

Tables structured *after* Szpak *et al.* (2017 Appendix A)

DA and DB correspond to 'duplicate A', and duplicate B', respectively.

$\delta^{13}C_{VPDB}$ and $\delta^{15}N_{AIR}$ correspond to normalised $\delta^{13}C$ and $\delta^{15}N$, respectively.

All values are rounded to 2 decimal places *after* Szpak *et al.* (2017).

Nb. One Seal standard (session 2 position 98) had an anomalous $\delta^{13}C$ value and was omitted from calculations lest this skew the results. The duplicate samples DA/DB_SED30C (session 3 positions 29 and 30) were for similar reasons also discarded because of considerable disparity in $\delta^{13}C$ values. Each of these anomalies is considered a machine error.

Session 1

Position	Sample	**Type** (standard type/sample)	Mass (mg)	$\delta^{13}C_{raw}$	$\delta^{15}N_{raw}$	$\delta^{13}C_{VPDB}$	$\delta^{15}N_{AIR}$	%C	%N	Atomic C:N
17	LEUCINE	Check	0.94	-28.27	6.56	-28.12	6.72	50.35	9.88	5.95
41	LEUCINE	Check	0.99	-28.20	6.27	-28.05	6.43	58.81	11.53	5.95
51	LEUCINE	Check	0.96	-28.29	6.24	-28.14	6.40	50.12	9.78	5.98
73	LEUCINE	Check	0.96	-28.31	6.16	-28.16	6.31	46.21	8.96	6.02
84	LEUCINE	Check	0.94	-28.03	6.30	-27.88	6.45	84.92	16.70	5.93
18	P2	Check	0.76	-28.19	-1.70	-28.04	-1.61	68.40	7.78	10.26
42	P2	Check	0.78	-28.14	-2.04	-27.99	-1.95	64.46	7.14	10.53
52	P2	Check	0.80	-28.17	-1.91	-28.02	-1.83	67.60	7.59	10.39
74	P2	Check	0.74	-28.15	-2.15	-28.00	-2.06	53.74	5.78	10.84
83	P2	Check	0.81	-28.01	-2.19	-27.86	-2.10	67.88	7.42	10.67
11	COW	Calibration	1.00	-24.53	7.69	-24.35	7.86	42.96	15.58	3.22
33	COW	Calibration	1.10	-24.58	7.82	-24.39	7.99	40.42	14.73	3.20
50	COW	Calibration	1.06	-24.47	7.78	-24.29	7.94	38.59	13.99	3.22
58	COW	Calibration	1.08	-24.54	7.82	-24.35	7.99	42.58	15.56	3.19
86	COW	Calibration	0.99	-24.45	7.79	-24.26	7.96	46.34	16.86	3.21
12	SEAL	Calibration	1.00	-12.81	15.93	-12.50	16.16	42.78	15.46	3.23
34	SEAL	Calibration	1.13	-12.91	15.93	-12.60	16.16	41.25	15.04	3.20
49	SEAL	Calibration	1.06	-12.84	15.89	-12.53	16.13	39.84	14.48	3.21
57	SEAL	Calibration	1.04	-12.87	15.75	-12.57	15.99	39.65	14.46	3.20
85	SEAL	Calibration	1.03	-12.77	15.79	-12.46	16.02	43.09	15.66	3.21
15	ALANINE	Calibration	1.03	-26.98	-1.56	-26.82	-1.47	41.28	16.02	3.01
23	ALANINE	Calibration	1.08	-26.98	-1.58	-26.82	-1.49	46.77	18.08	3.02
31	ALANINE	Calibration	1.11	-26.97	-1.66	-26.81	-1.57	42.63	16.38	3.04
39	ALANINE	Calibration	1.04	-27.23	-1.86	-27.07	-1.77	32.57	12.63	3.01
47	ALANINE	Calibration	1.15	-26.97	-1.59	-26.81	-1.50	40.89	15.74	3.03
55	ALANINE	Calibration	1.08	-27.11	-1.62	-26.95	-1.53	41.93	16.25	3.01
63	ALANINE	Calibration	1.06	-27.18	-1.85	-27.02	-1.76	37.70	14.66	3.00

Position	Sample	**Type** (standard type/sample)	Mass (mg)	$\delta^{13}C_{raw}$	$\delta^{15}N_{raw}$	$\delta^{13}C_{VPDB}$	$\delta^{15}N_{AIR}$	%C	%N	Atomic C:N
71	ALANINE	Calibration	1.00	-27.03	-1.73	-26.87	-1.64	36.83	14.26	3.01
79	ALANINE	Calibration	1.09	-27.08	-1.64	-26.92	-1.55	42.38	16.60	2.98
87	ALANINE	Calibration	1.11	-27.03	-1.67	-26.87	-1.58	41.75	16.25	3.00
92	ALANINE	Calibration	1.07	-27.02	-1.69	-26.85	-1.60	41.14	16.01	3.00
9	SED01A	Sample	2.59	-21.92	2.77	-21.72	2.89	41.74	2.55	19.08
10	SED01C	Sample	2.48	-21.00	1.57	-20.80	1.68	44.05	2.85	18.05
13	SED01E	Sample	2.62	-22.44	5.37	-22.31	5.49	44.72	4.46	11.71
14	SED01G	Sample	2.61	-21.36	0.84	-21.23	0.92	46.23	3.94	13.68
19	SED01J	Sample	2.59	-20.99	0.65	-20.77	0.73	45.68	3.22	16.54
20	DA_SED02A	Sample	2.65	-20.85	1.00	-20.63	1.07	47.40	3.44	16.07
21	DB_SED02A	Sample	2.64	-20.99	0.85	-20.78	0.91	46.32	3.42	15.79
22	SED02B	Sample	2.55	-21.14	0.72	-20.94	0.78	48.55	3.66	15.49
25	SED02H	Sample	2.60	-20.67	2.05	-20.46	2.17	49.55	5.07	11.40
26	SED02I	Sample	2.60	-20.98	4.42	-20.78	4.55	48.23	3.72	15.15
27	SED04A	Sample	2.59	-20.68	2.86	-20.49	2.99	50.20	4.44	13.18
28	SED04C	Sample	2.56	-20.90	4.14	-20.73	4.27	51.09	3.70	16.11
29	SED04G	Sample	2.50	-21.90	1.99	-21.76	2.11	36.67	2.21	19.35
30	SED04I	Sample	2.57	-19.68	2.45	-19.53	2.57	49.09	3.44	16.62
35	DA_SED05A	Sample	2.59	-22.30	2.29	-22.09	2.39	43.72	3.85	13.25
36	DB_SED05A	Sample	2.60	-22.18	1.96	-21.97	2.05	46.88	4.07	13.45
37	SED05B	Sample	2.65	-22.03	2.79	-21.82	2.88	46.57	4.01	13.56
38	SED05C	Sample	2.52	-21.32	-0.15	-21.11	-0.09	44.31	3.68	14.03
43	SED05D	Sample	2.60	-21.07	4.56	-20.82	4.76	45.82	4.44	12.05
44	SED05E	Sample	2.61	-21.11	3.75	-20.85	3.96	47.59	4.11	13.53
45	SED06A	Sample	2.58	-23.21	2.36	-22.97	2.58	37.11	2.39	18.11
46	SED06B	Sample	2.61	-23.74	7.46	-23.49	7.74	41.92	2.67	18.30
53	SED06F	Sample	2.56	-23.94	2.01	-23.78	2.12	46.54	2.22	24.44
54	SED06G	Sample	2.61	-24.68	1.88	-24.52	1.99	41.97	2.33	21.03
59	SED06I	Sample	2.56	-23.79	2.98	-23.64	3.09	44.41	2.42	21.43

Position	Sample	**Type** (standard type/sample)	Mass (mg)	$\delta^{13}C_{raw}$	$\delta^{15}N_{raw}$	$\delta^{13}C_{VPDB}$	$\delta^{15}N_{AIR}$	%C	%N	Atomic C:N
60	SED07A	Sample	2.57	-21.04	1.73	-20.87	1.81	29.20	1.55	21.91
61	SED07B	Sample	2.53	-20.36	3.28	-20.20	3.37	35.27	2.11	19.50
62	SED07C	Sample	2.54	-22.80	3.89	-22.68	3.99	35.90	2.05	20.45
65	SED07H	Sample	2.62	-21.94	2.46	-21.72	2.62	32.38	1.73	21.90
66	SED07I	Sample	2.52	-19.92	2.27	-19.68	2.46	38.32	2.85	15.69
67	DA_SED08A	Sample	2.61	-21.82	1.50	-21.59	1.71	36.08	1.87	22.46
68	DB_SED08A	Sample	2.54	-21.76	1.87	-21.52	2.12	36.01	1.83	22.90
69	SED08B	Sample	2.48	-20.24	1.42	-19.98	1.70	42.37	3.22	15.36
70	SED08F	Sample	2.64	-21.22	4.45	-20.96	4.79	40.25	2.30	20.45
75	SED08G	Sample	2.56	-20.95	4.88	-20.72	4.98	34.87	2.20	18.47
76	SED08I	Sample	2.53	-21.80	2.61	-21.57	2.68	29.80	1.56	22.25
77	DA_SED09A	Sample	2.53	-23.19	3.25	-22.97	3.31	38.78	2.15	21.00
78	DB_SED09A	Sample	2.62	-23.12	2.81	-22.90	2.86	43.30	2.47	20.48
81	SED09C	Sample	2.60	-22.83	3.78	-22.62	3.89	41.98	2.97	16.52
82	SED09D	Sample	2.57	-23.31	1.58	-23.11	1.66	43.68	1.83	27.88
89	DA_SED09F	Sample	2.51	-22.42	6.38	-22.22	6.57	43.95	3.31	15.48
90	DB_SED09F	Sample	2.57	-22.32	6.09	-22.13	6.30	44.05	3.23	15.92
91	SED09G	Sample	2.62	-24.37	0.73	-24.21	0.93	43.40	1.60	31.65

Session 2

Position	Sample	**Type** (standard type / sample)	Mass (mg)	$\delta^{13}C_{raw}$	$\delta^{15}N_{raw}$	$\delta^{13}C_{VPDB}$	$\delta^{15}N_{AIR}$	%C	%N	Atomic C:N
14	LEUCINE	Check	0.99	-28.19	6.69	-28.06	6.64	52.61	10.26	5.98
29	LEUCINE	Check	0.92	-28.02	6.51	-27.85	6.50	56.15	11.05	5.93
52	LEUCINE	Check	0.98	-28.31	6.64	-28.08	6.60	45.63	8.87	6.00
74	LEUCINE	Check	0.96	-28.28	6.66	-28.00	6.71	57.23	11.16	5.99
90	LEUCINE	Check	0.96	-28.32	6.43	-28.07	6.48	52.83	10.28	5.99
108	LEUCINE	Check	0.99	-28.26	6.54	-27.95	6.61	56.43	11.00	5.98
13	P2	Check	0.84	-27.97	-1.62	-27.83	-1.67	65.76	7.30	10.51
28	P2	Check	0.84	-27.95	-1.87	-27.77	-1.89	65.72	7.44	10.30
51	P2	Check	0.75	-28.08	-1.58	-27.85	-1.62	65.55	7.25	10.54
73	P2	Check	0.77	-28.16	-1.80	-27.90	-1.77	70.30	7.83	10.48
89	P2	Check	0.80	-28.11	-1.63	-27.86	-1.60	74.08	8.22	10.51
107	P2	Check	0.81	-28.00	-2.28	-27.72	-2.24	61.57	6.80	10.56
11	COW	Calibration	1.00	-24.37	7.93	-24.32	7.93	43.31	15.69	3.22
21	COW	Calibration	1.08	-24.34	7.72	-24.20	7.71	44.00	16.03	3.20
41	COW	Calibration	0.99	-24.42	8.00	-24.33	8.04	38.94	14.22	3.20
67	COW	Calibration	1.03	-24.44	7.92	-24.35	7.92	43.53	15.84	3.21
78	COW	Calibration	1.01	-24.49	7.79	-24.33	7.89	40.75	14.81	3.21
99	COW	Calibration	1.13	-24.47	8.00	-24.37	7.98	42.98	15.64	3.21
12	SEAL	Calibration	1.09	-12.64	16.13	-13.07	16.13	44.62	16.13	3.23
22	SEAL	Calibration	1.04	-12.73	15.95	-13.04	15.93	40.36	14.73	3.20
42	SEAL	Calibration	1.12	-12.89	16.12	-13.26	16.18	41.42	15.02	3.22
68	SEAL	Calibration	1.08	-12.65	16.18	-13.03	16.18	43.41	15.81	3.20
81	SEAL	Calibration	1.01	-12.84	16.10	-13.19	16.16	41.94	15.27	3.21
15	ALANINE	Calibration	1.14	-27.04	-1.46	-26.97	-1.54	40.07	15.55	3.01
23	ALANINE	Calibration	1.04	-27.07	-1.47	-26.82	-1.52	42.53	16.52	3.00
31	ALANINE	Calibration	1.08	-26.90	-1.60	-26.81	-1.65	41.29	15.99	3.01
39	ALANINE	Calibration	1.05	-26.99	-1.54	-26.79	-1.52	41.06	15.90	3.01

Position	Sample	**Type** (standard type / sample)	Mass (mg)	$\delta^{13}C_{raw}$	$\delta^{15}N_{raw}$	$\delta^{13}C_{VPDB}$	$\delta^{15}N_{AIR}$	%C	%N	Atomic C:N
47	ALANINE	Calibration	0.98	-27.03	-1.58	-26.91	-1.56	42.75	16.53	3.02
55	ALANINE	Calibration	1.03	-27.06	-1.66	-26.89	-1.78	36.41	14.14	3.00
63	ALANINE	Calibration	1.07	-27.03	-1.67	-26.76	-1.55	42.47	16.46	3.01
71	ALANINE	Calibration	1.15	-27.03	-1.58	-26.86	-1.66	42.61	16.50	3.01
79	ALANINE	Calibration	1.08	-27.11	-1.55	-26.85	-1.46	40.08	15.60	3.00
87	ALANINE	Calibration	1.00	-27.09	-1.66	-26.93	-1.64	42.65	16.47	3.02
95	ALANINE	Calibration	1.11	-27.09	-1.57	-26.92	-1.49	39.80	15.41	3.01
103	ALANINE	Calibration	1.12	-27.00	-1.51	-26.82	-1.62	41.80	16.14	3.02
111	ALANINE	Calibration	1.10	-27.07	-1.64	-26.78	-1.56	38.11	14.75	3.01
115	ALANINE	Calibration	1.04	-27.11	-1.61	-26.92	-1.56	38.08	14.75	3.01
9	SED10A	Sample	2.54	-20.73	4.02	-20.78	4.04	39.83	2.88	16.12
10	SED10D	Sample	2.60	-20.84	5.27	-20.91	5.28	42.90	4.54	11.03
17	SED10E	Sample	2.52	-22.83	3.88	-22.78	3.90	45.39	2.28	23.23
18	SED10G	Sample	2.65	-21.32	2.22	-21.32	2.23	44.87	2.14	24.43
19	SED10H	Sample	2.63	-20.53	5.37	-20.55	5.37	40.85	3.78	12.60
20	SED11A	Sample	2.60	-20.36	2.99	-20.38	2.98	44.35	4.97	10.42
25	SED11D	Sample	2.59	-21.58	2.40	-21.60	2.42	46.46	4.25	12.75
26	DA_SED11E	Sample	2.50	-19.60	3.58	-19.71	3.59	43.14	4.58	10.98
27	DB_SED11E	Sample	2.55	-19.58	3.65	-19.71	3.65	42.77	4.53	11.02
30	SED11G	Sample	2.56	-21.56	5.32	-21.67	5.29	47.61	3.35	16.59
33	SED11I	Sample	2.64	-19.77	7.14	-19.84	7.18	39.46	3.03	15.18
34	DA_SED12A	Sample	2.59	-24.05	0.62	-23.96	0.64	39.92	3.10	15.04
35	DB_SED12A	Sample	2.61	-24.02	1.22	-23.93	1.25	41.14	3.29	14.59
36	SED12B	Sample	2.58	-22.55	5.82	-22.52	5.86	46.45	5.81	9.33
37	SED12E	Sample	2.60	-18.97	10.77	-19.08	10.81	43.57	4.67	10.89
38	SED12F	Sample	2.53	-22.52	3.51	-22.50	3.54	37.14	2.28	18.97
43	SED12J	Sample	2.48	-22.66	5.07	-22.66	5.11	43.63	2.61	19.53
44	SED16A	Sample	2.59	-22.62	2.27	-22.63	2.30	47.92	2.02	27.74
45	SED16B	Sample	2.49	-22.48	3.72	-22.52	3.75	48.16	2.44	23.05

Position	Sample	Type (standard type / sample)	Mass (mg)	$\delta^{13}C_{raw}$	$\delta^{15}N_{raw}$	$\delta^{13}C_{VPDB}$	$\delta^{15}N_{AIR}$	%C	%N	Atomic C:N
46	SED16C	Sample	2.52	-19.19	11.11	-19.36	11.16	43.44	3.51	14.45
49	DA_SED16F	Sample	2.65	-22.61	0.89	-22.57	0.90	43.72	2.34	21.75
50	DB_SED16F	Sample	2.56	-22.59	0.85	-22.56	0.83	45.83	2.47	21.61
53	SED16H	Sample	2.53	-21.30	4.45	-21.34	4.38	46.86	2.49	21.96
54	SED17A	Sample	2.48	-21.07	2.59	-21.12	2.50	43.36	3.33	15.21
57	SED17B	Sample	2.51	-22.55	0.86	-22.50	0.90	45.63	2.67	19.91
58	DA_SED17C	Sample	2.54	-22.57	0.86	-22.51	0.91	46.80	2.60	21.01
59	DB_SED17C	Sample	2.50	-22.54	1.45	-22.48	1.52	47.49	2.66	20.83
60	SED17D	Sample	2.54	-19.95	3.74	-19.98	3.83	47.38	4.02	13.75
61	SED17E	Sample	2.62	-21.18	5.03	-21.15	5.14	43.92	5.73	8.95
62	SED18D	Sample	2.58	-22.15	4.67	-22.08	4.79	44.96	3.43	15.30
65	SED18E	Sample	2.65	-23.11	5.19	-23.05	5.21	37.12	1.83	23.69
66	SED18G	Sample	2.58	-22.74	8.03	-22.71	8.04	49.36	5.70	10.11
69	SED18I	Sample	2.54	-23.27	4.88	-23.23	4.84	44.36	2.71	19.09
70	SED19A	Sample	2.52	-22.03	5.07	-22.05	5.02	49.12	3.49	16.42
75	SED19B	Sample	2.65	-19.86	3.20	-19.91	3.26	47.10	3.03	18.14
76	SED19C	Sample	2.57	-20.33	7.79	-20.35	7.87	46.00	2.65	20.28
77	SED19D	Sample	2.58	-21.56	2.88	-21.53	2.96	50.11	2.65	22.09
82	DA_SED20C	Sample	2.61	-21.18	3.75	-21.21	3.78	46.01	2.90	18.50
83	DB_SED20C	Sample	2.64	-21.16	3.80	-21.20	3.83	46.36	2.85	19.01
84	SED20D	Sample	2.54	-20.92	3.38	-20.98	3.41	42.53	2.94	16.87
85	SED20E	Sample	2.56	-21.51	5.33	-21.55	5.37	43.53	4.02	12.63
86	SED20H	Sample	2.51	-23.03	3.13	-23.03	3.15	47.15	3.16	17.41
91	SED21C	Sample	2.58	-24.80	3.16	-24.69	3.21	45.56	1.90	28.02
92	SED21E	Sample	2.59	-21.81	3.60	-21.83	3.66	48.46	3.23	17.52
93	SED21G	Sample	2.63	-23.42	4.27	-23.38	4.34	47.41	1.96	28.16
94	SED21H	Sample	2.65	-23.98	4.76	-23.92	4.84	44.46	1.90	27.29
97	SED18J	Sample	2.57	-24.51	5.17	-24.40	5.18	37.49	1.64	26.70
100	SED19H	Sample	2.54	-20.61	5.21	-20.66	5.17	43.93	3.21	15.99

**Position**	**Sample**	**Type** (standard type / sample)	**Mass (mg)**	$\delta^{13}C_{raw}$	$\delta^{15}N_{raw}$	$\delta^{13}C_{VPDB}$	$\delta^{15}N_{AIR}$	**%C**	**%N**	**Atomic C:N**
101	DA_SED20G	Sample	2.50	-21.43	3.34	-21.46	3.27	46.09	3.52	15.28
102	DB_SED20G	Sample	2.66	-21.45	3.23	-21.48	3.14	40.20	3.10	15.14
105	SED28J	Sample	2.52	-20.43	12.28	-20.46	12.34	45.38	2.56	20.69
106	SED29J	Sample	2.61	-21.49	-0.31	-21.47	-0.27	42.71	5.05	9.88
109	SED30I	Sample	2.61	-23.09	3.34	-22.98	3.41	44.98	2.53	20.73
110	SED31D	Sample	2.61	-20.89	0.53	-20.85	0.61	39.07	2.71	16.83
113	SED32I	Sample	2.57	-22.29	1.94	-22.26	1.98	43.54	2.97	17.08
114	SED33E	Sample	2.59	-23.52	2.15	-23.46	2.20	46.34	2.21	24.49

Session 3

Position	Sample	Type (standard type / sample)	Mass (mg)	$\delta^{13}C_{raw}$	$\delta^{15}N_{raw}$	$\delta^{13}C_{VPDB}$	$\delta^{15}N_{AIR}$	%C	%N	Atomic C:N
18	LEUCINE	Check	0.97	-28.28	6.88	-28.07	7.01	52.15	10.26	5.93
35	LEUCINE	Check	0.98	-28.23	6.59	-28.03	6.72	56.06	11.02	5.93
52	LEUCINE	Check	0.94	-28.40	6.63	-28.23	6.76	49.96	9.73	5.99
61	LEUCINE	Check	0.92	-28.41	6.65	-28.10	6.73	54.53	10.67	5.96
17	P2	Check	0.82	-28.08	-1.72	-27.88	-1.63	62.89	7.17	10.23
34	P2	Check	0.84	-28.03	-1.66	-27.83	-1.57	67.16	7.63	10.28
46	P2	Check	0.77	-28.00	-1.79	-27.88	-1.60	71.14	7.91	10.50
60	P2	Check	0.80	-28.20	-1.55	-27.92	-1.49	68.42	7.65	10.43
11	COW	Calibration	1.08	-24.35	7.66	-24.21	7.85	44.92	16.31	3.21
19	COW	Calibration	1.09	-24.62	7.93	-24.41	8.07	40.98	14.97	3.19
27	COW	Calibration	1.13	-24.55	7.75	-24.33	7.91	32.68	11.86	3.21
58	COW	Calibration	1.09	-24.53	7.93	-24.27	8.05	43.42	15.89	3.19
12	SEAL	Calibration	0.98	-12.67	15.71	-12.53	15.96	44.28	16.16	3.20
20	SEAL	Calibration	1.02	-12.82	15.87	-12.58	16.05	39.41	14.41	3.19
33	SEAL	Calibration	1.15	-12.72	15.90	-12.48	16.08	41.08	14.98	3.20
59	SEAL	Calibration	1.03	-12.85	16.07	-12.55	16.22	41.64	15.19	3.20
15	ALANINE	Calibration	1.02	-26.93	-1.81	-26.90	-1.59	46.30	17.92	3.01
23	ALANINE	Calibration	1.08	-27.13	-1.62	-26.92	-1.54	39.06	15.16	3.01
31	ALANINE	Calibration	1.06	-27.10	-1.85	-26.89	-1.72	41.61	16.12	3.01
39	ALANINE	Calibration	1.13	-27.00	-1.68	-26.81	-1.60	43.41	16.80	3.01
47	ALANINE	Calibration	1.15	-27.09	-1.68	-26.98	-1.48	42.37	16.45	3.01
55	ALANINE	Calibration	1.14	-27.16	-1.62	-27.02	-1.53	41.70	16.25	2.99
63	ALANINE	Calibration	1.11	-27.22	-1.59	-26.87	-1.56	40.74	15.82	3.00
67	ALANINE	Calibration	1.14	-27.04	-1.75	-26.81	-1.74	40.39	15.63	3.01
9	SED28B	Sample	2.61	-22.51	4.91	-22.31	5.06	49.72	2.90	20.00
10	SED28D	Sample	2.58	-21.18	2.44	-21.01	2.59	52.33	3.22	18.96
13	SED28E	Sample	2.65	-20.82	5.10	-20.73	5.32	46.79	3.66	14.91

Position	Sample	Type (standard type / sample)	Mass (mg)	$\delta^{13}C_{raw}$	$\delta^{15}N_{raw}$	$\delta^{13}C_{VPDB}$	$\delta^{15}N_{AIR}$	%C	%N	Atomic C:N
14	SED28G	Sample	2.65	-20.74	3.34	-20.67	3.57	44.74	3.08	16.97
21	DA_SED29C	Sample	2.51	-22.09	1.53	-21.87	1.63	42.62	3.27	15.19
22	DB_SED29C	Sample	2.65	-22.05	1.58	-21.83	1.68	43.02	3.38	14.86
25	SED29D	Sample	2.52	-22.34	4.94	-22.12	5.07	42.10	3.93	12.49
26	SED29H	Sample	2.63	-19.85	1.32	-19.63	1.44	43.47	4.40	11.53
28	SED29I	Sample	2.65	-22.04	3.47	-21.82	3.62	45.32	3.93	13.46
36	SED30D	Sample	2.65	-22.24	3.89	-22.02	4.00	45.30	2.76	19.17
37	SED30E	Sample	2.64	-24.88	4.51	-24.68	4.63	43.15	2.16	23.30
38	SED30F	Sample	2.62	-22.47	4.30	-22.26	4.41	45.32	3.00	17.62
41	SED031A	Sample	2.65	-20.52	3.20	-20.31	3.33	42.58	3.71	13.38
42	DA_SED31B	Sample	2.49	-23.25	0.85	-23.06	0.99	46.10	2.14	25.11
43	DB_SED31B	Sample	2.60	-23.20	1.11	-23.03	1.26	46.93	2.13	25.66
44	SED31C	Sample	2.55	-21.62	2.06	-21.46	2.24	47.00	2.15	25.47
45	SED31F	Sample	2.65	-22.89	3.54	-22.74	3.74	46.33	1.89	28.65
49	DA_SED32C	Sample	2.65	-19.82	2.36	-19.60	2.48	48.60	4.53	12.51
50	DB_SED32C	Sample	2.55	-19.77	2.32	-19.57	2.44	49.00	4.52	12.65
51	SED32E	Sample	2.62	-21.44	0.77	-21.25	0.87	44.68	2.84	18.39
53	SED32F	Sample	2.59	-22.11	6.63	-21.94	6.76	43.25	2.73	18.50
54	SED32H	Sample	2.57	-20.93	1.34	-20.77	1.45	46.95	3.16	17.34
57	SED33A	Sample	2.57	-23.40	2.79	-23.16	2.89	43.14	2.06	24.43
62	SED33B	Sample	2.64	-22.53	2.75	-22.19	2.80	41.83	2.05	23.82
65	SED33G	Sample	2.64	-24.02	3.59	-23.80	3.68	44.65	1.97	26.51
66	SED33J	Sample	2.60	-22.58	2.70	-22.35	2.76	50.71	2.53	23.37
